# Regulation of rose petal dehydration tolerance and senescence by *RhNAP* transcription factor via the modulation of cytokinin catabolism

**DOI:** 10.1186/s43897-021-00016-7

**Published:** 2021-10-11

**Authors:** Jing Zou, Peitao Lü, Liwei Jiang, Kun Liu, Tao Zhang, Jin Chen, Yi Yao, Yusen Cui, Junping Gao, Changqing Zhang

**Affiliations:** 1 Department of Ornamental Horticulture, China Agricultural University, Beijing, 100193 China; 2 College of Horticulture, FAFU-UCR Joint Center for Horticultural Biology and Metabolomics, Haixia Institute of Science and Technology, Fujian Agriculture and Forestry University, Fuzhou, 350002 China

**Keywords:** *Rosa hybrida*, RhNAP, RhCKX6, Dehydration, Petal senescence, Cytokinins

## Abstract

Petals and leaves share common evolutionary origins but have different phenotypic characteristics, such as the absence of stomata in the petals of most angiosperm species. Plant NAC transcription factor, NAP, is involved in ABA responses and regulates senescence-associated genes, and especially those that affect stomatal movement. However, the regulatory mechanisms and significance of NAP action in senescing astomatous petals is unclear. A major limiting factor is failure of flower opening and accelerated senescence. Our goal is to understand the finely regulatory mechanism of dehydration tolerance and aging in rose flowers. We functionally characterized *RhNAP*, an *AtNAP*-like transcription factor gene that is induced by dehydration and aging in astomatous rose petals. Cytokinins (CKs) are known to delay petal senescence and we found that a cytokinin oxidase/dehydrogenase gene 6 (*RhCKX6*) shares similar expression patterns with *RhNAP*. Silencing of *RhNAP* or *RhCKX6* expression in rose petals by virus induced gene silencing markedly reduced petal dehydration tolerance and delayed petal senescence. Endogenous CK levels in *RhNAP*- or *RhCKX6*-silenced petals were significantly higher than those of the control. Moreover, *RhCKX6* expression was reduced in *RhNAP*-silenced petals. This suggests that the expression of *RhCKX6* is regulated by *RhNAP*. Yeast one-hybrid experiments and electrophoresis mobility shift assays showed that RhNAP binds to the *RhCKX6* promoter in heterologous in vivo system and in vitro, respectively. Furthermore, the expression of putative signal transduction and downstream genes of ABA-signaling pathways were also reduced due to the repression of PP2C homolog genes by RhNAP in rose petals. Taken together, our study indicates that the *RhNAP/RhCKX6* interaction represents a regulatory step enhancing dehydration tolerance in young rose petals and accelerating senescence in mature petals in a stomata-independent manner.

**Supplementary Information:**

The online version contains supplementary material available at 10.1186/s43897-021-00016-7.

## Core

Cytokinins (CKs) play an important role in the regulation of environmental stress responses and organ senescing processes through keeping at the appropriate level of CK activity. Here we reveal that the dehydration- and aging-induced *RhNAP* physically binds to the promoter of *RhCKX6*, promoting CK catabolism. The RhNAP/RhCKX6 interaction represents a regulatory step enhancing dehydration tolerance in young rose petals and accelerating senescence in mature petals in a stomata-independent manner.

## Introduction

Plant organ senescence is a finely tuned developmental process during which the constituent cells undergo dramatic changes in metabolism, structure and gene expression (Woo et al. 2018; Ma et al. 2018) and, ultimately, programmed cell death (PCD) (van Doorn and Woltering 2008; Kabbage et al. 2017). Developmentally regulated senescence has been studied in leaves (Zhang and Gan 2012; Jiang et al. 2014), fruits (Jiang et al. 2017) and flowers (Wu et al. 2017; Lü et al. 2014), and it has also been shown that environmental conditions, such as drought, darkness, high temperature and salinity, as well as pathogen challenge, can trigger organ senescence (Sade et al. 2018; Patharkar and Walker 2019). However, the mechanistic relationships between senescence that is developmentally programmed or that which is environmentally induced, or the nature of any shared signaling pathways, are not well understood (Guo and Gan 2012).

It has been reported that senescence programs in both leaves and flowers involve the preferential expression of a specific set of senescence associated genes (SAGs), which include regulatory transcription factors (TFs) and structural proteins (Gao et al. 2016; Li et al. 2018; Sun et al. 2021). Among these, different classes of TF genes have been functionally associated with leaf and/or flower senescence, including MYB, MYC, C2H2-type zinc-finger, AP2/EREBP, MADS-Box, NAC and WRKY domain TFs, as well as homeodomain proteins (Li et al. 2018; Shahri and Tahir 2014). As an example, the *A. thaliana* NAC family gene *NAC-LIKE*, *ACTIVATED BY AP3*/*PI* (*AtNAP*) is substanially upregulated in senescing leaves, and it has been found that *AtNAP* overexpression results in precocious leaf, while loss-of-function mutants show significantly delayed senescence (Guo and Gan 2006). AtNAP is also known to act together with a protein phosphatase 2C (PP2C) to mediate the abscisic acid (ABA) regulated promotion of stomatal opening and water loss in senescing leaves, as part of the ABA-AtNAP-SAG113 PP2C signal transduction pathway (Zhang and Gan 2012). *AtNAP* homologs have also been identified in rice (*O. sativa*) (Liang et al. 2014) and morning glory (*I. nil*) (Shinozaki et al. 2014).

In addition to TFs, senescence is also controlled by the contrasting actions of a number of phytohormones (Sakuraba et al. 2014). For example, while ethylene and ABA promote senescence, it is substantially delayed by treatment with cytokinins (CKs). In addition to influencing organ and whole plant senescence, CKs regulate many physiological and developmental processes, including cell division (cytokinesis), nutrient mobilization, shoot apical meristem activity, floral development, chloroplast development and differentiation (Bartrina et al. 2011). CK biosynthesis is controlled by the ATP/ADP isopentenyltransferase (IPT) genes, which encode rate-limiting enzymes in CK biosynthesis. Conversely, CK degradation is mediated by cytokinin oxidase/dehydrogenase (CKX), which irreversibly degrades active CKs into adenine or adenosine and side chains (Frébort et al. 2011; Hwang et al. 2012). Changes in the expression levels of CK metabolism genes presumably help maintain the appropriate level of CK activity; however, the means by which this control is exerted during developmentally or environmentally controlled senescence is not known.

CKs are known to delay flower senescence in a number of important ornamental species, including carnation (*D. caryophyllus*) (Eisinger 1977), petunia (*P. hybrida*) (Taverner et al. 1999) and rose (*Rosa hybrida*) (Mayak and Halevy 1970, 1974). An inverse relationship between CK content and senescence has been identified floral tissues (Van Staden et al. 1988) and the importance of CK levels for flower senescence is also suggested by a number of other observations. For example, the longevity of petunia flowers was greatly extended in plants expressing a bacterial *IPT* gene that provides precursors for CK biosynthesis, under the control of the senescence-associated *SAG12* promoter (Chang et al. 2003). Moreover, the exogenous application of 6-methylpurine, a CKX inhibitor, was reported to substantially increase the life span of carnation petals (Taverner et al. 2000). A reported increase in the mRNA abundance of two *CKX* genes during carnation petal senescence is also suggestive of accelerated CK breakdown (Hoeberichts et al. 2007). Such observations indicate a function for CKs in delaying petal senescence, but the means by which CK levels are regulated has not been established.

There is increasing evidence that CKs play an important role in the regulation of environmental stress responses, involving intensive interactions and crosstalk with ABA (Verslues 2016; Nishiyama et al. 2011; Verma et al. 2016). CKs and ABA exert antagonistic activities during several developmental and physiological processes, including plant adaptation to environmental stresses, stomatal closure and leaf senescence (Gan and Amasino 1995; Chang et al. 2003; Nishiyama et al. 2011). When plants are exposed to stress, the accumulation of ABA specifically promotes stomatal closure to minimize water loss and accelerates leaf senescence. Conversely, CKs trigger responses to delay both stomatal closure and leaf senescence (Pospisilova 2003; Pospisilova et al. 2005), although it is worth noting that differences in drought related phenotypes are not always associated with stomata-related traits (Bartels and Sunkar 2005; Fujita et al. 2005; Hirayama and Shinozaki 2010). For instance, the *AREB1ΔQT* transgenic *A. thaliana* plants showed enhanced drought tolerance but no differences in stomatal movement or function (Fujita et al. 2005).

Shared regulation mechanisms exist between CK and ABA metabolism and signaling during different processes that involve plant adaptation to stresses, as well as plant growth and development (Nishiyama et al. 2011). However, the molecular pathways that govern the antagonistic actions of CK and ABA in organ senescence and dehydration tolerance are still unclear, particularly in petals that have no stomata. Similarly, while NAP is involved in ABA responses and regulates senescence-associated genes, and especially those that affect stomatal movement (Zhang and Gan 2012; Liang et al. 2014; Hu et al. 2021), the regulatory mechanisms and significance of NAP action in senescing astomatous petals is unclear (van Doorn 1997).

In this current study we isolated a dehydration- and senescence-induced *AtNAP*-like gene, *RhNAP*, from rose petals. We found that *RhNAP* shares similar expression patterns with a rose cytokinin oxidase/dehydrogenase gene (*RhCKX6*) in senescing petals or those undergoing dehydration. Silencing of *RhNAP* or *RhCKX6* expression decreased young petal dehydration tolerance and delayed mature petal senescence. RhNAP was found to physically bind to the *RhCKX6* promoter both in vivo and in vitro. Together with ABA signaling cascade mediated by RhNAP-RhPP2C interactions, we propose that RhNAP-RhCKX6 associations regulate petal dehydration tolerance and senescence in rose flowers.

## Results

### *RhNAP* and *RhCKX6* are co-expressed in response to dehydration and during petal senescence

Based on our previous microarray results (Dai et al. 2012), we identified a dehydration-induced unigene, *JK619941*, encoding a rose NAC family TF. Phylogenetic analysis and protein sequence alignments showed that this NAC protein is closely related to *A. thaliana* AtNAP (Fig. S1) and so the unigene was named *RhNAP*. A GAL4 transient expression assay of RhNAP, also in *Arabidopsis* protoplasts, indicated that RhNAP functions as a transcriptional activator with a transactivation domain at the C-terminus (Fig. S2). Our previous microarray analysis also indicated the involvement of CK metabolism in the dehydration response and a *CYTOKININ OXIDASE/DEHYDROGENASE* (*CKX*) gene (*JK618028*) was observed to be highly up-regulated during dehydration (Dai et al. 2012). Phylogenetic analysis indicated that this gene is a homolog of CKX genes from *F. vesca* (*FvCKX6*) and *A. thaliana* (*AtCKX6*) (Fig. S3). We designated *JK618028* as *RhCKX6*.

**Fig. 1:**
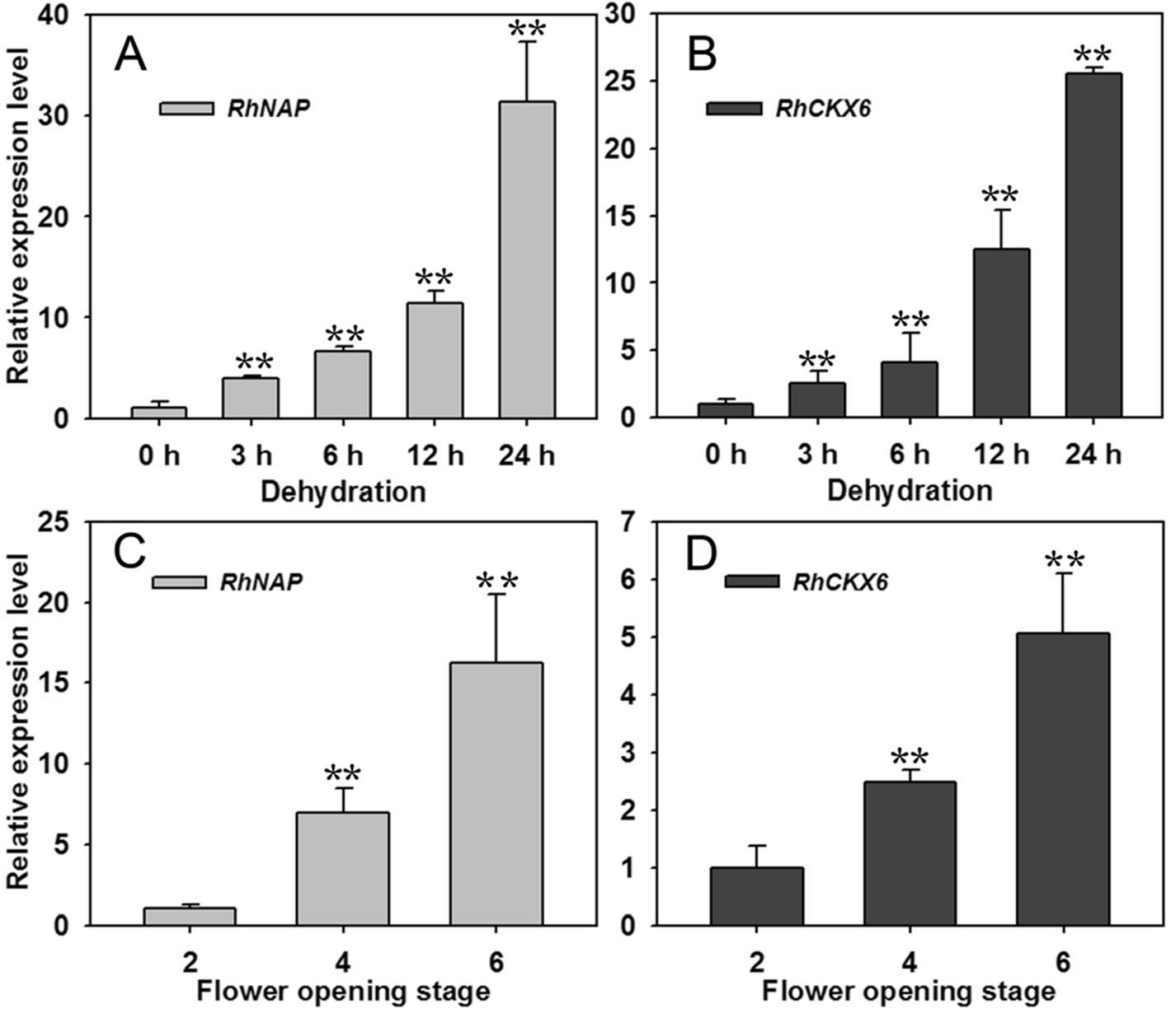
Both *RhNAP* and *RhCKX6* are induced by dehydration and petal senescence. qRT-PCR analysis of *RhNAP* and *RhCKX6* expression in rose petals in response to dehydration treatment (**A, B**). qRT-PCR analysis of *RhNAP* and *RhCKX6* expression in rose petals at different opening stages (**C, D**). *RhUBI1* was used as an internal control. All data shown are means ± standard deviation (*n* = 3); Student’s *t*-test, *P < 0.05, **P < 0.01

We further confirmed the expression profiles of both *RhNAP* and *RhCKX6* in rose petals subjected to a dehydration treatment by quantitative RT-PCR assays. The transcript levels of *RhNAP* and *RhCKX6* increased substantially in rose petals after 3 h of dehydration and were 31-fold and 26-fold greater, respectively, than levels in control petals after 24 h of dehydration (Fig. 1A, B). Dehydration typically results in ABA accumulation in rose petals (Le Page-Degivry et al. 1991), so we also tested the expression levels of *RhNAP* and *RhCKX6* in petals following treatment with ABA. Both genes were express at higher levels in ABA treated petals than in controls (Fig. S4).

*AtNAP* is a key regulator of leaf senescence (Guo and Gan 2006; Zhang and Gan 2012) and dehydration stress can trigger the senescence of plant organs (Zhang and Gan 2012). We therefore assessed the expression of *RhNAP* and *RhCKX6* during petal senescence. qRT-PCR showed that the expression levels of both genes increased substantially in rose petals in parallel with flower aging from a full opened flower (opening stage 4) to the onset of petal wilting (opening stage 6), supporting a functional association of both genes with petal senescence (Fig. 1C, D).

### Functional analysis of *RhNAP* and *RhCKX6* in association with dehydration tolerance in rose petals and *A. thaliana* seedlings

**Fig. 2:**
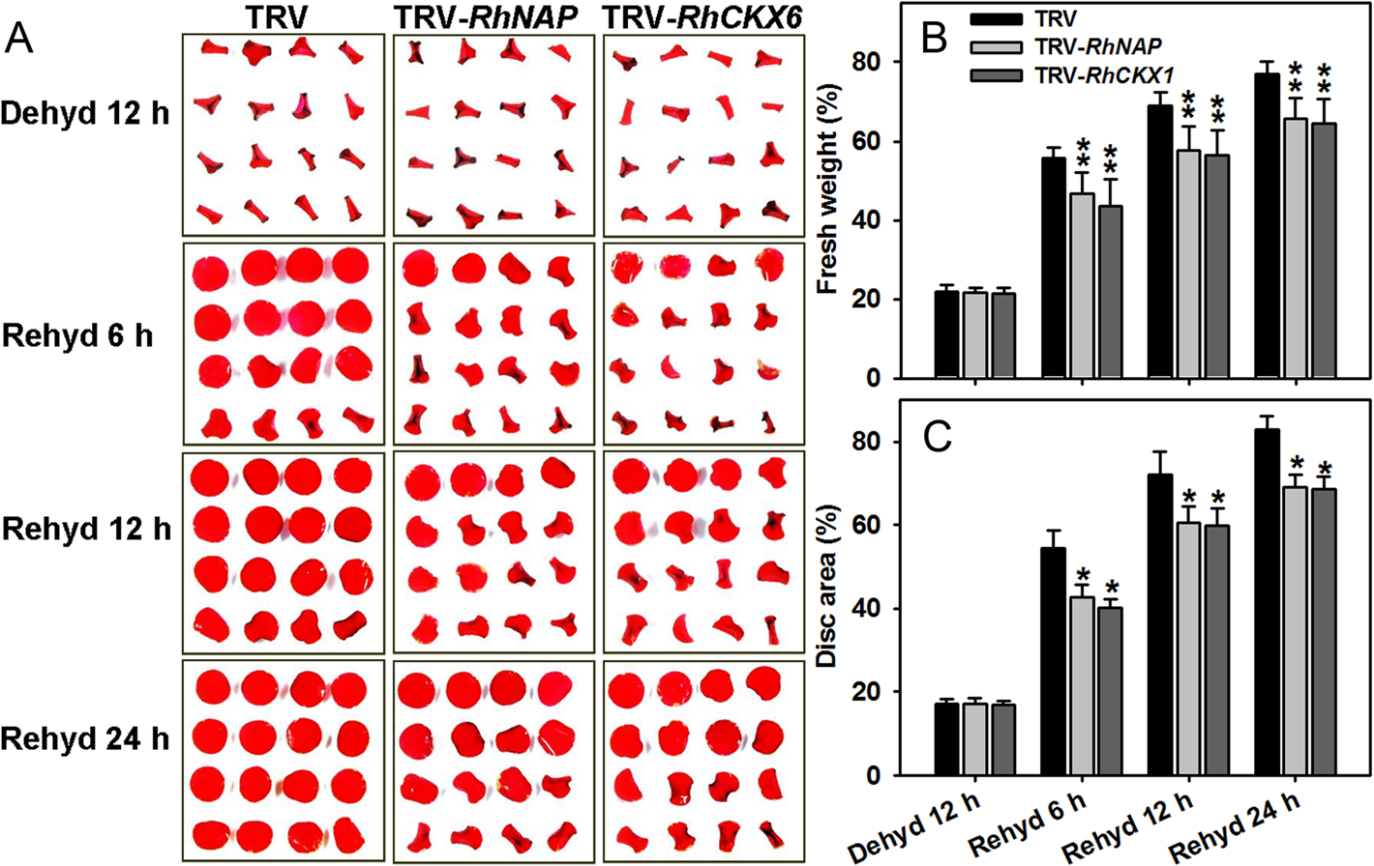
*RhNAP-* or *RhCKX6*-silencing reduced dehydration tolerance in rose petal discs. Expression of *RhNAP* and *RhCKX6* was silenced in petal discs by virus induced gene silencing (VIGS). Petal discs were dehydrated for 12 h and examined at intervals during 24 h of rehydration. **A** The phenotypes of the petal discs were recorded and photographed at different time points. The fresh weight (**B**) and recovery area (**C**) of the petal discs were determined. *n* = 5 ± standard deviation values are shown in (**B**) and (**C**); Student’s *t*-test, *P < 0.05, **P < 0.01. Dehyd, dehydration; Rehyd, rehydration

The dehydration tolerance of rose petals can be assessed by evaluating the expansion of intact petals or petal discs after rehydration, as described by Dai et al. (2012). In this current study, we similarly used petal disc fresh weight and expansion area to determine the potential roles of *RhNAP* and *RhCKX6* in rose petal dehydration, after suppressing the expression of each gene in petal discs using virus-induced gene silencing (VIGS). The *RhNAP*- or *RhCKX6*-specific 3′ end regions were used to construct tobacco rattle virus vectors (TRV-*RhNAP* and TRV-*RhCKX6*, respectively) to enable specific gene silencing. Petal discs of rose flowers (stage 2) were dehydrated for 12 h and then rehydrated for 24 h (Fig. 2A). After 6 h of rehydration, the fresh weight of 56% of the discs derived from the TRV control petals had recovered, compared with only 47 and 44% of those from *RhNAP-* and *RhCKX6*-silenced petals, respectively (Fig. 2B). The differences between TRV control and gene silenced discs were still significant after 24 h of rehydration. Additionally, the areas of the expanded discs were significant decreased in *RhNAP-* and *RhCKX6*-silenced discs compared with those of the TRV treated control (Fig. 2C). These results suggest that *RhNAP* and *RhCKX6* are involved in dehydration tolerance in young rose petals.

To further examine the function of *RhNAP* and *RhCKX6* in dehydration tolerance, we overexpressed *RhNAP* and *RhCKX6* separately in *A. thaliana* and subjected the two-week old seedlings grown in soil to drought stress. After 15 d of drought treatment, the plants were allowed to recover under normal growth conditions for 3 d. More than 60% of the *RhNAP*-ox and *RhCKX6*-ox *Arabidopsis* plants survived compared with only 32% of the controls (Fig. S5). Thus, *RhNAP* and *RhCKX6* can confer drought stress tolerance when expressed heterologously in *A. thaliana* seedlings.

### Functional association of *RhNAP* and *RhCKX6* with rose petal senescence

**Fig. 3:**
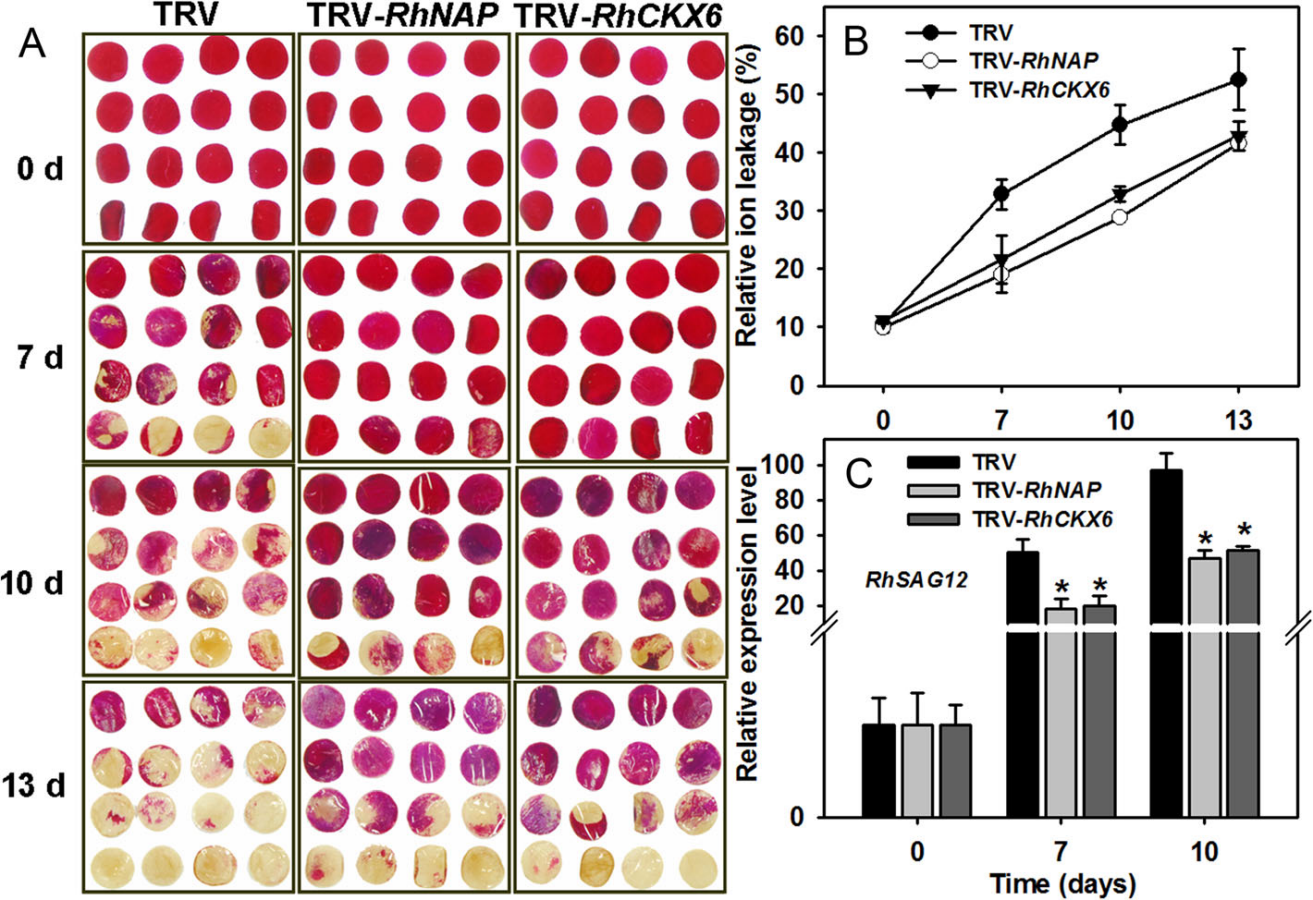
*RhNAP*- or *RhCKX6*-silencing delays senescence in rose petal discs. *RhNAP* and *RhCKX6* expression was silenced in petal discs by virus induced gene silencing (VIGS). **A** The phenotypes of the petal discs were recorded and photographed at various time points (days) until necrosis was observed. The ion leakage (**B**) and relative expression of *RhSAG12* by qRT-PCR (**C**) were determined. *n* = 3 ± standard deviation values are shown in (**B**) and (**C**); Student’s *t*-test, *P < 0.05

Both NAP TFs and CKs have previously been associated with petal senescence: *AtNAP* was reported to be upregulated in senescing *A. thaliana* petals (Wagstaff et al. 2009), as was also the case with a gene homolog in senescing morning glory petals (Shinozaki et al. 2014), while CKs have been reported to delay rose flower senescence (Mayak and Halevy 1970, 1974). We first investigated the effects of different types of CKs on the rose petal senescence and determined that the application of all those tested (6-BA, *t*Z and iP) delayed the senescence of petal discs, while the CK inhibitor lovastatin promoted their senescence (Fig. S6). We hypothesized that the senescence-induced *RhNAP* and *RhCKX6* genes may be involved in rose petal senescence and tested this using *RhNAP*- and *RhCKX6*-silenced petal discs. We found that suppressing *RhNAP* and *RhCKX6* expression indeed delayed petal disc senescence compared with TRV-treated controls (Fig. 3A), and that ion leakage was also significantly reduced compared to the control discs (Fig. 3B). Additionally, *RhSAG12* transcript levels were significantly down-regulated in *RhNAP*- and *RhCKX6*-silenced discs compared with those of the TRV-treated control (Fig. 3C).

To further test the putative functional association between *RhNAP* and *RhCKX6* and senescence, we expressed each gene separately in *A. thaliana*, generating *RhNAP*-ox and *RhCKX6*-ox transgenic lines, respectively. As shown in Fig. S7, plants from both genotypes had smaller leaves and age equivalent transgenic plants (approximately 35 d after germination, or DAG) exhibited precocious leaf senescence phenotypes compared with the controls.

### Expression of *RhCKX6* is predominantly dependent on *RhNAP*

**Fig. 4:**
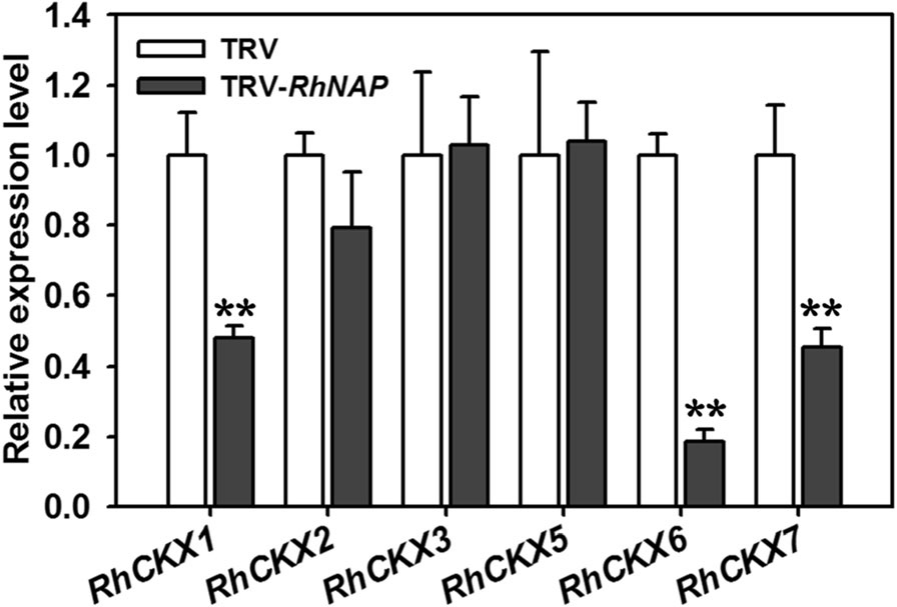
Transcript levels of *RhCKX* genes in *RhNAP*-silenced petals. Expression of *RhNAP* in petals was silenced by virus induced gene silencing (VIGS). Petals were sampled for qRT-PCR analysis of *RhCKX* gene expression and *RhUBI1* was used as an internal control. All data shown are means ± standard deviation (*n* = 3); Student’s *t*-test, *P < 0.05, **P < 0.01

Our results showed that *RhNAP* and *RhCKX6* exhibit similar expression patterns under dehydration conditions and during senescence, yield similar phenotypes when silenced in petal discs, and confer similar degrees of enhanced drought tolerance and leaf senescence when overexpressed in *A. thaliana*. To test the hypothesis that *RhCKX6* operates downstream of *RhNAP* action in rose petals, we examined the effect of silencing of *RhNAP* on the expression of six rose CKX (*RhCKX*) genes. We observed that the expression level of *RhCKX6* was reduced to 20% of control levels in *RhNAP*-silenced petals (Fig. 4). In addition, *RhCKX1* and *RhCKX7*, two close homologs of *RhCKX6*, were also clearly down-regulated in the *RhNAP*-silenced petals compared with TRV controls (Fig. 4). However, *RhCKX1* and *RhCKX7* were not induced by ABA (Fig. S4) or dehydration treatments, or as a result of aging (Fig. S8*A* and *B*), and so are likely not regulated by *RhNAP* during petal dehydration and senescence. Taken together these data suggest that the dehydration- and senescence-upregulated *RhCKX6* gene is likely regulated by the RhNAP TF.

### RhNAP binds to the *RhCKX6* promoter

**Fig. 5:**
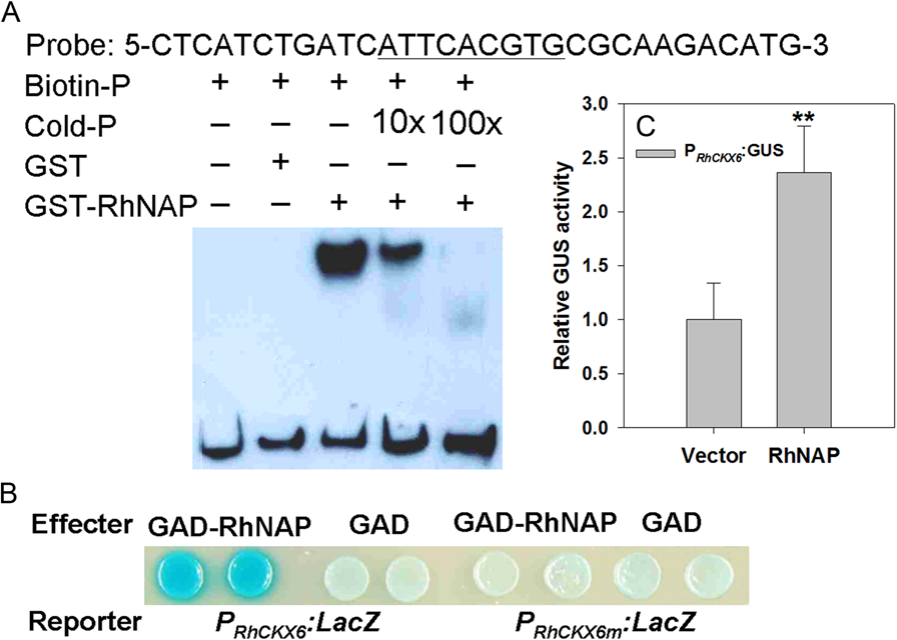
RhNAP binding to *cis*-elements in the promoter of *RhCKX6*. **A** Wild-type and mutant probes derived from the *RhCKX6* promoter. The wild-type *cis*-element and its nucleotide substitutions in the mutants are underlined. Interaction between GST-RhNAP and the biotin-labeled probe on a native PAGE gel. Purified protein (3 μg) was incubated with 25 pM of the biotin-labeled wild-type probe. Non-labeled probe with different concentrations (from 10 to 100 x) was added for the competition test. **B** Transactivation activity of RhNAP with the *RhCKX6* promoter in yeast. GAD-RhNAP, but not GAD itself, activates expression of the *LacZ* reporter gene driven by the wild-type 31-bp fragment of the *RhCKX6* promoter. The mutated fragment abolishes activation of the *LacZ* reporter gene expression. **C** Regulation of the *RhCKX6* promoter activity by RhNAP in *A. thaliana* mesophyll protoplasts. The effector constructs contained GFP-RhNAP or GFP alone, driven by the super1300 promoter. The reporter constructs contained the *RhCKX6* promoter (− 1308 bp to − 1 bp upstream of ATG). Protoplasts were co-transformed with different combinations of effector and reporter constructs and the relative GUS activity indicated the promoter activity. Normalized GUS activities are presented as the means ± standard deviation (*n* = 6). The difference was statistically significant (Student’s *t*-test, *P* < 0.01) as denoted by asterisks

To test the hypothesis that RhNAP directly regulates *RhCKX6* expression in rose petals, we performed a gel-shift assay to determine whether the RhNAP protein binds to the *RhCKX6* promoter. A 1308-bp putative promoter region immediately upstream of the *RhCKX6* coding sequence was amplified and a 31-bp fragment spanning positions − 558 to − 528 of the *RhCKX6* promoter was used as probe (Fig. 5A). The probe contains the 9-bp sequence 5’ATTCACGTG3’, which contains a predicted NAC recognition site CGT[G/A] (Tran et al. 2004; Franco-Zorrilla et al. 2014), and the reverse complementary sequence of this segment (5’CACGTGAAT3’) is very similar to AtNAP core binding sequence (5’CACGTAAGT3’) (Zhang and Gan 2012). A recombinant form of the RhNAP protein fused to the C terminus of glutathione S-transferase, (GST)-RhNAP, was expressed in and purified from *E. coli*, then co-incubated and electrophoresed with the biotin-labeled and/or non-labeled probe. As shown in Fig. 5A, a shifted DNA-binding band was detected with addition of GST-RhNAP and labeled DNA probes, but no band was detected in the GST control. When unlabeled DNA probe concentrations were gradually increased in the reaction mixture, the DNA-binding signal gradually weakened.

To test the interaction of RhNAP with the *RhCKX6* promoter in vitro*,* we performed a yeast one-hybrid assay (Fig. 5B). The *RhCKX6 cis*-element promoter fragment and its corresponding mutant, 5’CGGACATGT3’, were each used to drive the *LacZ* reporter gene (Fig. 5B). The *RhNAP* open reading frame (ORF) was fused to the yeast GAL4 activation domain (GAD) to generate the effector construct GAD-RhNAP. The yeast one-hybrid experiment confirmed that RhNAP can indeed bind to the *RhCKX6* promoter baits, but not to the GAD promoter or the *RhCKX6* mutant fragment. These results indicate that RhNAP is capable of directly promoting *RhCKX6* expression through binding to a 9-bp *cis*-element sequence, 5’ATTCACGTG3′ in the *RhCKX6* promoter.

We also tested the effects of RhNAP action on *RhCKX6* expression in *A. thaliana* protoplasts. When both the Super1300:RhNAP effector construct and the P_*RhCKX6*_:GUS reporter construct were introduced into the protoplasts, GUS activity was nearly 2.5-fold greater than that of the controls (Fig. 5C).

### Cytokinin contents of *RhNAP*-silenced rose petals

**Fig. 6:**
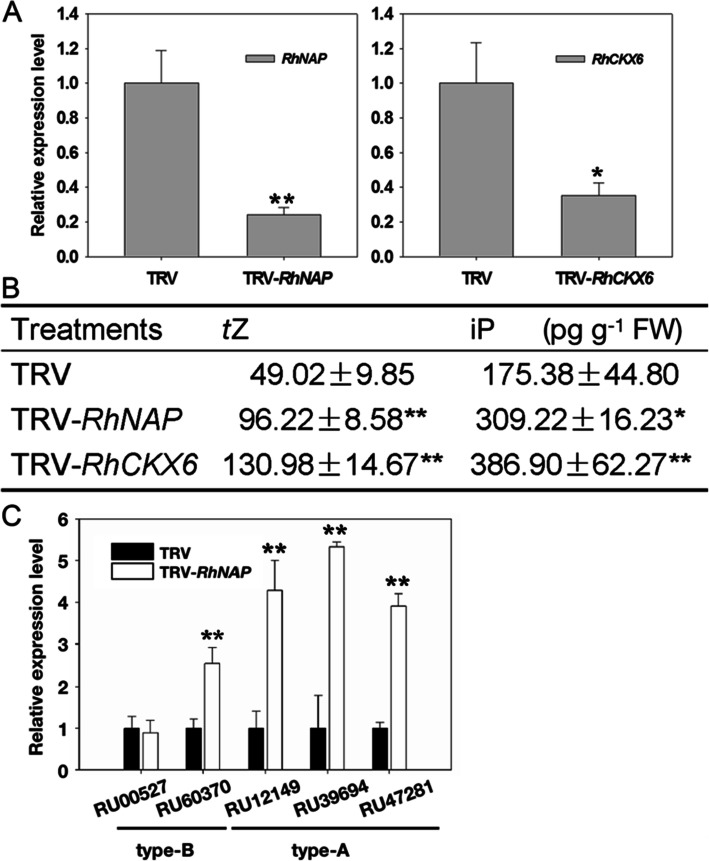
Endogenous cytokinins levels in TRV, TRV-*RhNAP* and TRV-*RhCKX6* treated petals. **A** The relative expression levels of *RhNAP* and *RhCKX6* in silenced rose petal discs by qRT-PCR. *RhUBI1* was used as the internal control. **B** The cytokinin quantification analysis. FW, fresh weight. **C** Expression analysis of type-A (*RU12149, RU39694, RU47281*) and type-B *(RU00527 and RU60370*) *response regulator* (*RR*) genes in *RhNAP*-silenced petals by qRT-PCR. *RhUBI1* was used as an internal control. *n* = 3 ± standard deviation values are shown; Student’s *t*-test, *P < 0.05, **P < 0.01

It is well established that CKX enzymes catalyze the breakdown of CKs (Frébort et al. 2011), so we analyzed of *RhNAP* or *RhCKX6* expression in silenced petal discs, respectively (Fig. 6A), and then measured endogenous CK levels in TRV, TRV-*RhNAP* and TRV-*RhCKX6* treated petal discs. Levels of the CKs *trans*-zeatin (*t*Z) and N^6^-(Δ^2^-isopentenyl) adenine (iP) in TRV-*RhNAP* were 196, and 176%, respectively, of those detected in TRV control discs, while the abundance of *t*Z and iP in TRV-*RhCKX6* treated discs were 267 and 221%, respectively, those of the controls (Fig. 6B). Thus, silencing of either *RhCKX6* or *RhNAP* resulted in increased levels of CKs, further suggesting the regulation of *RhCKX6* activity by RhNAP.

Two types (type A and B) of functional response regulators (RRs) are involved in CK signaling in order to activate the transcription of CK responsive genes (Kieber and Schaller 2018). To gain further insights into the molecular processes associated with the elevated levels of CKs in *RhNAP*-silenced petals, we investigated the transcript levels of RR genes in the petals. Five RR genes were selected, comprising three type-A (*RU12149*, *RU39694*, *RU47281*) and two type-B (*RU00527* and *RU60370*). Other than *RU00527,* the expression of the RR genes was significantly up-regulated in the *RhNAP*-silenced petals (Fig. 6C). These results suggest that *RhNAP-RhCKX6* interaction controls CK steady state levels and subsequent downstream CK signaling during petal dehydration tolerance and senescence.

### RhNAP activates the ABA signaling cascade in rose petals

**Fig. 7:**
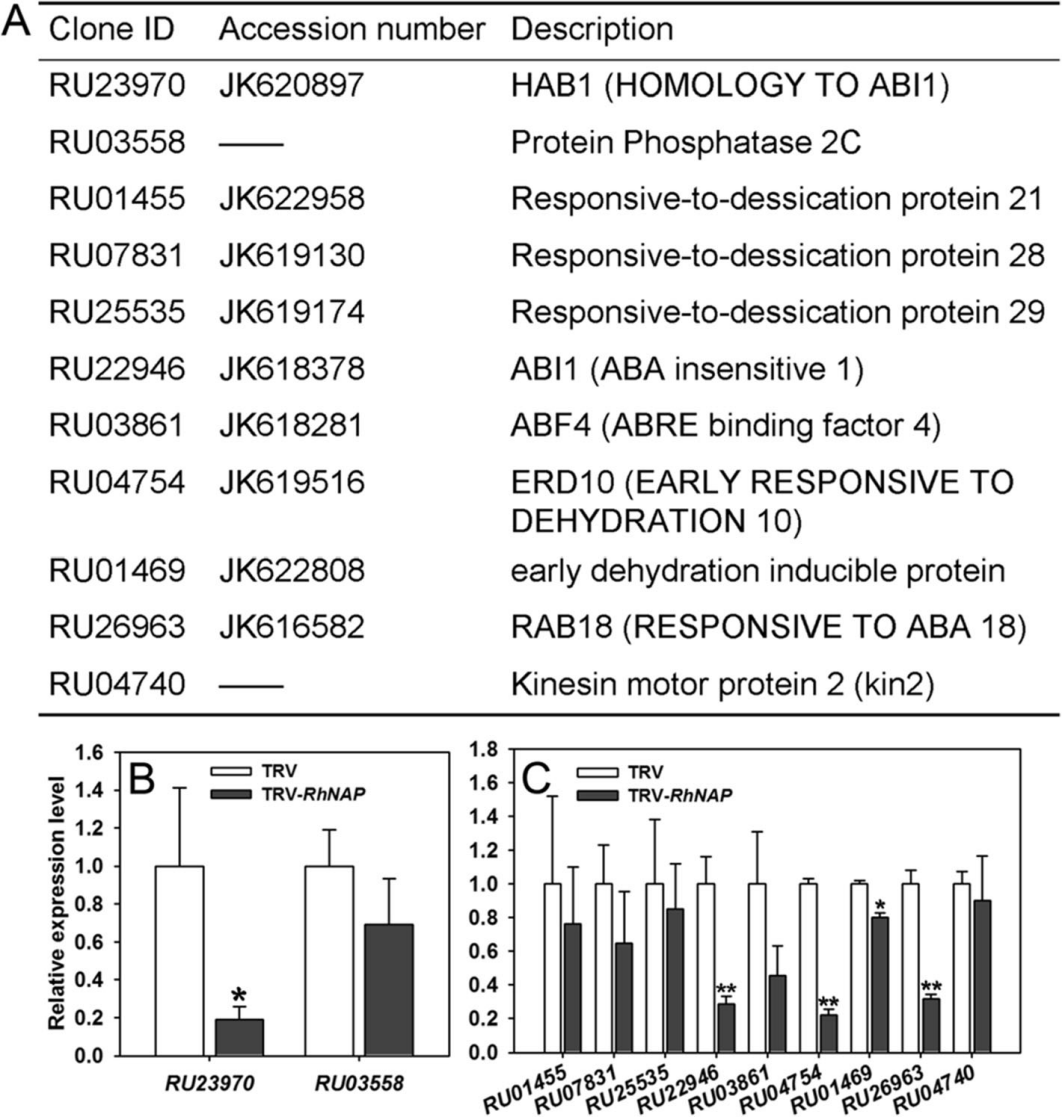
Expression of *PP2C* genes and other ABA-related genes in *RhNAP*-silenced rose petals. **A** The putative ABA signaling and downstream rose genes from the ABA-signaling pathway. The clone IDs correspond to the rose transcriptome database (Dai et al. 2012). The description of the *A. thaliana* homolog is as given by The Arabidopsis Information Resource (TAIR, http://www.arabidopsis.org). **B** qRT-PCR analysis of two *PP2C* genes in *RhNAP*-silenced rose petals. **C** The expression patterns of 9 ABA responsive genes in *RhNAP*-silenced rose petals were analyzed by qPCR. The internal control used was *RhUBI1*. All data shown are means ± standard deviation (*n* = 3); Student’s *t*-test, *P < 0.05, **P < 0.01

AtNAP has been reported to be involved in regulating leaf senescence and stomatal movement by influencing the action of *SAG113*, a PP2C family PP through an ABA-RhNAP-SAG113 regulatory chain (Zhang and Gan 2012). Since we determined that *RhNAP* is also induced by ABA (Fig. S4), we reasoned that RhNAP might regulate the expression of PP2C family genes in rose petals. We therefore assessed the expression of two *PP2C* genes that we had previously identified from our rose transcriptome databases (Dai et al. 2012; Pei et al. 2013), a rose homolog of *SAG113* (*RU03558*) and a dehydration-induced *PP2C* gene (*RU23970*) (Fig. 7A), in *RhNAP*-silenced rose petals. We found that both genes were down-regulated in *RhNAP*-silenced petals, and that *RU23970* showed a particularly low expression level of (Fig. 7B). This suggested that the ABA signaling pathway is also involved in RhNAP-mediated functions during petal dehydration tolerance. We therefore evaluated the expression of 9 additional rose genes putatively involved in ABA-signaling pathways (Fig. 7A). qRT-PCR analysis revealed that the expression levels of 6 of these genes, including the rose homologs of *RD21*, *RD28*, *ABI1*, *ABF4*, *ERD10* and *RAB18*, were clearly repressed in RhNAP-silenced rose petals (with a fold change < 0.8) (Fig. 7C). These data suggest RhNAP activates ABA-responsive gene expression via the ABA signaling pathway, thereby enhancing the dehydration tolerance of rose petals.

## Discussion

### RhNAP integrates the signals of dehydration- and aging-induced petal senescence in rose flower

Plant-specific NAC (NAM/ATAF1,2/CUC2) TFs play important roles in regulating diverse biological processes, including development, senescence, growth, cell division and responses to environmental stresses (Olsen et al. 2005). Comparative analysis of *A. thaliana* gene expression has shown that some NAC TFs are up-regulated in senescent petals (Wagstaff et al. 2009). NAP (NAC-LIKE, ACTIVATED BY AP3/PI), a member of the NAC subfamily, was reported to function in the transition between growth by cell division and by cell expansion in stamens and petals (Sablowski and Meyerowitz 1998). Later reports showed that the *NAP* genes are mainly involved in organ senescence, such as *AtNAP* in leaves, siliques and flowers (Guo and Gan 2006; Zhang and Gan 2012), *OsNAP* in leaves of rice (Liang et al. 2014), and the morning glory gene *InNAP* in flowers (Shinozaki et al. 2014). In this current study, we identified a dehydration-induced rose NAC gene, *RhNAP*, whose expression is also induced during petal aging (Fig. 1C) and by ABA treatments (Fig. S4). Furthermore, silencing of *RhNAP* expression decreased the tolerance to dehydration and delayed the senescence of petal discs (Figs. 2 and 3).

As well as being developmentally controlled, the onset of senescence can also be induced by unfavorable environmental conditions, such as water deficit (Sade et al. 2018; Patharkar and Walker 2019; Guo and Gan 2012). In the context of senescence, expression profiling of *A. thaliana* TFs has revealed that a considerable number are induced during senescence and are also upregulated by various stresses, suggesting extensive overlap between senescence and stress responses (Chen et al. 2002; Li et al. 2018). Here we found that RhNAP both enhances dehydration tolerance in young rose petals and promotes petal senescence. Moreover, *RhNAP*-overexpressing *A. thaliana* seedlings showed improved drought tolerance while the mature plants exhibited precocious senescence (Fig. S5 and S7). We interpret these results to suggest that RhNAP integrates the signals of developmental senescence and dehydration-induced senescence in rose petals at juvenile stages.

### RhNAP is involved in petal dehydration tolerance and senescence through promotion of *RhCKX6* expression

CKs regulate numerous biological processes, including organ senescence and responses to environmental stresses via complex metabolic and signaling networks (Frébort et al. 2011; Ha et al. 2012; Hwang et al. 2012; Kieber and Schaller 2018; Hönig et al. 2018). It is well established that CKX enzymes degrade active CKs into adenine or adenosine and side chains; however, less is known about the regulation of the corresponding genes. In rose petals, we found that the CKX gene, *RhCKX6* was induced by both dehydration treatments and aging (Fig. 1B and D). Silencing of *RhCKX6* expression decreased petal disc dehydration tolerance and delayed their senescence, as was observed with silencing of *RhNAP* (Figs. 2 and 3). Moreover, we found that the expression of *RhCKX6* was reduced in *RhNAP*-silenced petals (Fig. 4). We observed that RhNAP can bind to a 9 bp ATTCACGTG segment of the *RhCKX6* promoter, as revealed using EMSA method (Fig. 5A), and that it can activate the *RhCKX6* promoter in yeast and *A. thaliana* protoplasts (Fig. 5B and C). Furthermore, the CK contents of both *RhNAP*-silenced and *RhCKX6*-silenced petals were shown to be higher than those of the TRV control (Fig. 6B). Interestingly, we also found that *RhCKX1* and *RhCKX7* expression was reduced in *RhNAP*-silenced petals and 6-BA treated petals. However, these two genes were not induced by ABA (Fig. S4) or dehydration treatments, or as a consequence of aging (Fig. S8*A* and *B*). We suggest that *RhCKX1* and *RhCKX7* are not directly regulated by *RhNAP*, but that their reduced expression resulted from the high levels of CKs in *RhNAP*-silenced petals (Fig. 6B), and we note that CKs have been reported to upregulate the expression of multiple *CKX* genes in *A. thaliana* (Nishiyama et al. 2011) and rice (Raines et al. 2016). Based on these results, the *RhNAP/RhCKX6* interaction is proposed as a key step in the regulation of dehydration tolerance and senescence in rose petals.

### RhNAP functions in dehydration tolerance and senescence via stomata-independent pathways in rose petals

Petals and leaves share common evolutionary origins but perform very different functions and, accordingly, their physiologies and gene expression profiles have common but distinct patterns during their senescence (Price et al. 2008; Wagstaff et al. 2009). An ABA-AtNAP-SAG113 PP2C regulatory chain has been proposed to regulate leaf senescence by controlling stomatal movement and water loss in *A. thaliana* (Zhang and Gan 2012). In rice leaves, *OsNAP* is induced by ABA, and its expression is reduced in the ABA biosynthetic mutants *aba1* and *aba2* (Liang et al. 2014). We determined in this current study that *RhNAP* expression is induced by dehydration and ABA in astomatous rose petals (Fig. 1A, S4 and S9), and that its silencing results in reduced expression of two predicted *PP2C* genes (*RU03558* and *RU23970)* (Fig. 7B). In addition, several putative ABA-signaling pathway genes were also down-regulated (Fig. 7C), which we propose contributed to the observed decreased dehydration tolerance of *RhNAP-*silenced young petals. These observations are congruent with several reports that variation in plant drought phenotypes is not always related to stomatal function (Bartels and Sunkar 2005; Fujita et al. 2005; Hirayama and Shinozaki 2010).

Changes in endogenous CK levels have been reported to alter the stress tolerance of plants (Rivero et al. 2007; Havlova et al. 2008; Nishiyama et al. 2011) and prolonged drought has been associated with a reduction in active CK levels (Nishiyama et al. 2011), growth reduction and reallocation of limited energy resources towards defense against environmental stresses. Here, elevated CKs concentrations that resulted from silencing *RhNAP* or *RhCKX6* expression were also correlated with reduced dehydration tolerance in young rose petal discs (flower opening stage 2) (Figs. 2 and 6B). Conversely, decreased CKs levels in *RhNAP*- or *RhCKX6*-overexpressing *A. thaliana* transgenic plants were associated with enhanced drought stress tolerance of the young seedlings (Fig. S5). Interestingly, while microarray and RT-PCR experiments have demonstrated that *A. thaliana CKX1*, *CKX3*, *CKX4* and *CKX6* are down-regulated by ABA (Werner et al. 2006), we observed that ABA treatment increased the expression of *RhCKX* genes in rose petals (Fig. S4), as did dehydration (Dai et al. 2012). We hypothesize that this may be due to the stressed young rose petals having high CK activity (Mayak and Halevy 1970).

CKs are known to delay the senescence of vegetative and floral organs (Van Staden et al. 1988). For example, elevated CK levels delayed flower petal senescence in several ornamental plants, including carnations (Taverner et al. 2000; Hoeberichts et al. 2007), petunia (Taverner et al. 1999; Chang et al. 2003), and roses (Mayak and Halevy 1970, 1974). In our study, the concentration of CKs increased and the expression levels of CK signaling and responsive genes were upregulated, which we propose contributed to the observed delayed senescence of *RhNAP*- or *RhCKX6-*silenced petal discs (Figs. 3 and 6). This is consistent with the observation that leaf senescence is associated with a decrease in CK content and CK signaling suppression (Gan and Amasino 1995; Kim et al. 2006; Hu et al. 2021).

**Fig. 8:**
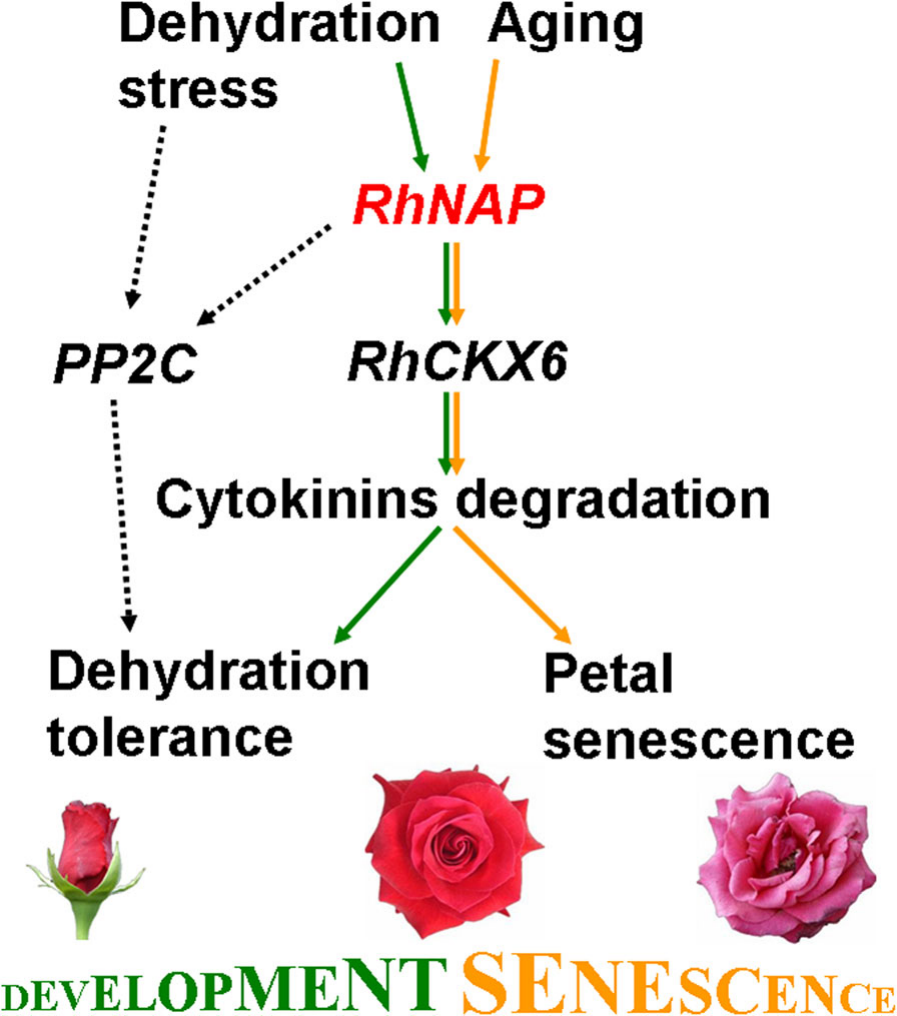
RhNAP regulating rose petal dehydration tolerance and senescence by modulating the degradation of CKs. Dehydration stress and aging induce the expression of *RhNAP*, which directly activates the cytokinin oxidase/dehydrogenase gene 6 (*RhCKX6*), resulting in the degradation of endogenous CKs, thereby enhancing dehydration tolerance in young petals and promoting petal senescence. The *PP2C* genes also participate in the up-regulation of petal dehydration tolerance via *RhNAP* action. Green lines indicate the signaling pathway for dehydration stress via the *RhNAP/RhCKX6* regulatory point in young rose petals, while yellow lines indicate aging through the *RhNAP/RhCKX6* regulatory step in mature rose petals. Dashed lines indicate steps operating via the ABA signaling pathway or possible unidentified components

In summary, we have shown that dehydration- and aging-induced RhNAP expression modulates dehydration tolerance and senescence in rose petals and that *RhCKX6* functions as a direct downstream target of *RhNAP*. The RhNAP integrates the signals of developmental senescence and dehydration-induced senescence in rose petals at juvenile stages. We conclude that the *RhNAP/RhCKX6* regulatory interaction promotes dehydration tolerance in young rose petals and accelerates petal senescence via stomata-independent mechanisms (Fig. 8).

## Methods

### Plant material and growth conditions

Flowers of *Rosa hybrida* (cv. ‘Samantha’) were harvested at the different opening stages (Ma et al. 2005), placed in water and delivered to the laboratory within 1 h. Stems were re-cut under water to ~ 25 cm length and uniform flowers were selected and kept in deionized water until further processing. Petal discs were taken from the same whorl of petals from flowers at opening stage 2 and were immersed in solutions containing different CKs, including 6-benzylaminopurine (6-BA, 100 μM), *trans*-zeatin (*t*Z, 10 μM), isopentenyladenine (iP, 10 μM), or the CK biosynthesis inhibitor lovastatin (20 μM). Control samples were treated with 0.05% EtOH without any phytohormones.

*A. thaliana* ecotype Col-0 seeds were sterilized and sown on Murashige and Skoog (MS) salts medium and appropriate antibiotics, then kept at 4 °C for 3 d to allow germination. Approximately 7-day old seedlings were transplanted into pots containing a 1:1 mixture of vermiculite and peat moss. Seedlings were grown at 22 °C and 50% relative humidity under a 16/8 h light/dark photoperiod.

### Cloning, plasmid construction and plant transformation

The ORFs and promoter sequences of *RhNAP* and *RhCKX6* were amplified using SMART™ RACE cDNA Amplification kit (Clontech, Palo Alto, CA, USA). All PCR products were subcloned into the pGEM T-Easy Vector (Promega, Madison, WI, USA) and then transformed into *E. coli* DH5*a* cells and sequenced. All primer sequences used in this study are listed in Table S1.

For construction of the *RhNAP* VIGS vector, a 347-bp fragment at the 3′ end of *RhNAP* was amplified. The PCR products were digested with *Bam*HI and *Xho*I and ligated into the corresponding sites of the pTRV2 vector (Dai et al. 2012) to generate the pTRV2-*RhNAP* construct. For the construction of the *RhCKX6* VIGS vector, a 228-bp fragment from the 3’ end of the gene was used.

In order to express the RhNAP recombinant protein in *E. coli* for EMSA assays, the RhNAP ORF was amplified by PCR and subcloned into the *Bam*HI and *Eco*RI sites of the pGEX-2 T vector (GE Healthcare, Piscataway, NJ, USA), allowing the production of the GST-RhNAP fusion protein. The GST tag in pGEX-2 T was used to facilitate the purification of the fusion protein.

For the yeast one-hybrid assay, the *RhNAP* ORF was cloned into the *Eco*RI and *Xho*I sites of the pJG4–5 vector (Clontech) to produce the GAD-RhNAP construct. To generate the *LacZ* reporter genes driven by the *RhCKX6* promoter with a wild-type or mutant motif, 35-bp oligonucleotides were synthesized. The annealed oligonucleotides were ligated into the *Eco*RI and *Xho*I sites of pLacZi2μ (Lin et al. 2007), generating the constructs P_*RhCKX6*_:*LacZ* and P_*RhCKX6m*_:*LacZ*, respectively.

To generate the GUS reporter gene driven by the *RhCKX6* promoter (P_*RhCKX6*_:GUS), a 1308-bp fragment upstream of *RhCKX6* ATG was PCR amplified and cloned into the binary pBI121 vector (Clontech) to replace the 35S CaMV promoter.

To generate the *RhNAP* and *RhCKX6* overexpression binary vectors, The ORFs of *RhNAP* and *RhCKX6* were amplified by PCR and the resulting fragments were inserted into a modified binary pCAMBIA 1300 vector harboring a super promoter (Super1300) and a green fluorescent protein (GFP) encoding sequence (Gong et al. 2002). The resulting constructs (Super1300:RhNAP and Super1300:RhCKX6) were transformed into *Agrobacterium* strain GV3101 and then introduced into *A. thaliana* plants via the floral dip method (Clough and Bent 1998).

The *Arabidopsis* mesophyll protoplasts were isolated as described in Yoo et al. (2007). The plasmids were extracted from transformed *E. coli* DH5*α* cells using the MACHEREY-NAGEL nucleic acid purification kit (MACHEREY-NAGEL, Düren, Germany), then 10 μg effector plasmid (Super1300:RhNAP) and 10 μg reporter plasmid (P_*RhCKX6*_:GUS) were transformed into 100 μl protoplasts containing ~ 2 × 10^4^ protoplasts by polyethylene glycol mediated transformation.

For the transactivation assay in yeast (*S. cerevisiae*), different portions of RhNAP to be examined were PCR amplified using forward primers with the *Sal*I site at the 5′ end and reverse primers with the *Pst*I site at the 5′ end. The amplified fragments were digested with *Sal*I and *Pst*I and inserted in frame into the *Sal*I and *Pst*I sites of the pBD vector (Clontech) to make expression vectors. The proteins fused with pBD-GAL4 are as follows: pBD-RhNAPF (1–282 of RhNAP), pBD-RhNAPN (1–160 of RhNAP), pBD-RhNAPC (161–282 of RhNAP).

### Silencing of *RhNAP* and *RhCKX6* in rose petals by VIGS

Silencing of *RhNAP* and *RhCKX6* expression by VIGS was performed as described by Dai et al. (2012), with some minor modifications. The pTRV1, pTRV2, pTRV2-*RhNAP* and pTRV2-*RhCKX6* vectors were transformed into the *A. tumefaciens* strain GV3101, and the transformed *A. tumefaciens* lines were cultured for 24 h in Luria-Bertani (LB) medium supplemented with 20 mM acetosyringone, 50 μg ml^− 1^ kanamycin and 50 μg ml^− 1^ gentamycin sulfate. The cultures were harvested and suspended in infiltration buffer (10 mM MgCl_2_, 200 mM acetosyringone and 10 mM MES, pH 5.6) to a final OD_600_ of approximately 1.8. A mixture of cultures containing an equal ratio (v/v) of pTRV1 and pTRV2, pTRV1 and pTRV2-*RhNAP* or pTRV1 and pTRV2-*RhCKX6*, were used as TRV control, TRV-*RhNAP* and TRV2-*RhCKX6*, respectively. The mixtures were placed at room temperature in the dark for 4 h before vacuum infiltration of rose petals. Petals from the middle whorl at flower stage 2 were collected and 1 cm diameter discs were excised from the center of the petals with a hole punch. Vacuum infiltration was performed by immersing rose petals or discs in the bacterial suspension solution and infiltrating under a vacuum at 0.7 MPa. After release of the vacuum, petals and discs were washed in deionized water and kept in deionized water for 3 d at 8 °C, followed by an equilibrium step at 23 °C for 1 d. For RNA extraction, petals were kept in deionized water at 23 °C until sampling. For determination of dehydration tolerance, petal discs were dehydrated for 12 h and examined at intervals during 24 h of rehydration. The senescing phenotypes of petal discs were observed daily until necrosis was observed.

### Extraction and quantification of endogenous cytokinins

Endogenous cytokinins (CKs) were extracted from rose petals and quantified as described in Pan et al. (2010). Petal disc material (approximately 100 mg) was frozen in liquid nitrogen, ground to fine powder and extracted with extraction solvent (2-propanol:H_2_O:concentrated HCl [2:1:0.002 v/v/v]; sample:solvent = 1:10 mg μl^− 1^) on a shaker (100 rpm) at 4 °C for 30 min. One milliliter of dichloromethane was added to each sample, and the samples were shaken (100 rpm) for 30 min at 4 °C. After centrifugation (13,000 *g*, 4 °C, 5 min), two phases were present and the lower phase (~ 1.5 ml) was collected. The solvent mixture was concentrated to near dryness using a concentrator (Eppendorf, Hamburg, Germany) and then redissolved in 0.1 ml methanol. The sample solution was centrifuged at 12,000 *g* for 5 min and then analyzed HPLC-electrospray ionization-tandem mass spectrometry (HPLC-ESI-MS/MS). The extracts were analyzed by multiple reaction monitoring (MRM) on an Agilent 1260 Infinity HPLC System (Agilent, Santa Clara, CA, USA) coupled via ESI source to a QTrap 5500 System (AB Sciex, Foster City, CA, USA). A 10 μl aliquot of solution was injected, and analyzed on an Agilent SB-C18 (4.6 mm id, 50 mm length, 1.8 μm C18 resin, Agilent) at 30 °C. Eluent A was acetonitrile, and eluent B consisted of a 0.1% acetic acid aqueous solution. A gradient elution with the following composition was used: 10% A at 0 min, 90% A at 5 min. The flow rate was 0.8 ml min^− 1^. Data acquisition and processing were performed with AB Analyze software (AB Sciex).

### Ion leakage quantification

To measure relative electrolyte leakage, petal samples at each time point were placed in a 50-ml tube containing 20 ml of deionized water and incubated at 25 °C for 30 min on an orbital shaker (200 rpm). The initial conductivity of the fluid was measured with a conductivity detector (Shanghai INESA, Shanghai, China). The samples were then boiled for 10 min in deionized water and cooled to room temperature. The total conductivity was then determined as before, and the relative electrolyte leakage was expressed as the percentage of the initial conductivity versus total conductivity.

### Quantitative RT-PCR

For qRT-PCR analysis, 1 μg DNase treated total RNA was used to synthesize cDNA according to the manufacturer’s instructions using a reverse transcription system A3500 kit (Promega), with a 20 μl reaction volume. A 2 μl aliquot of cDNA was used as the template in a 20 μl qRT-PCR reaction using the Applied Biosystems StepOnePlus™ real-time PCR system (Applied Biosystems, CA, USA) with KAPA™ SYBR® FAST qPCR kits (Kapa biosystems, Boston, MA, USA). All reactions were performed with three biological replicates. Relative gene expression values were calculated according to the 2^−ΔΔCT^ method, in which *RhUBI1* (GenBank accession JK622648) was used as an internal control (Meng et al. 2013).

### Purification of recombinant protein and EMSA

EMSA assays were performed as previously described (Lü et al. 2014). Briefly, the GST-RhNAP fusion protein was induced in 100 ml cultures of the transformed *E. coli* BL21 cells by adding isopropyl β-D-1-thiogalactopyranoside (IPTG) to a final concentration of 0.2 mM and the cultures were incubated at 28 °C for 6 h. The recombinant protein was purified using Glutathione Sepharose 4B beads (GE Healthcare, Piscataway, NJ, USA) according to the manufacturer’s instructions. EMSA was performed using the LightShift chemiluminescent EMSA kit (Pierce, IL, USA), according to the manufacturer’s instructions. Briefly, the biotin-labeled DNA fragments (5′-CTCATCTGATCATTCACGTGCGCAAGACATG-3′) were synthesized, annealed and used as probes, with unlabeled DNA of the same sequence used as a competitor.

### Yeast one-hybrid assay

Yeast one-hybrid assays were performed as described by Lin et al. (2007). Briefly, a plasmid containing the GAD-RhNAP fusion sequence was co-transformed with different *LacZ* reporter gene constructs into the yeast strain EGY48 as described in the Yeast Protocols Handbook (Clontech). Transformants were grown on SD/−Trp-Ura dropout plates containing 80 mg L^− 1^ X-gal (5-bromo-4-chloro-3-indolyl-β-D-galactopyranoside) and the color development of yeast colonies was observed.

### Supplementary Information

**Additional file 1: Figure S1.** Analysis of the RhNAP protein sequence. (*A*) Phylogenetic analysis of RhNAP together with known NAC family proteins. The phylogenetic tree file was produced by MEGA 5.2. Bootstrap values indicate the divergence of each branch and the scale indicates branch length. Accession numbers are as follows: ATAF1 (AT1G01720), ATAF2 (AT5G08790), ANAC019 (AT1G52890), ANAC032 (AT1G77450), ANAC055 (AT3G15500), ANAC072 (AT4G27410), ANAC102 (AT5G63790), OsNAC6 (BAA89800), TaNAC69 (AAY44098.1), GmNAC11 (ACC66315.1), GmNAC20 (ACC66314), AtNAP (AT1G69490), PhNAP (AAM34773), InNAP (AB639146), OsNAP (LOC_Os03g21060), RhNAP (JK619941), RhNAC2 (JK619963), RhNAC3 (JK617768), RhNAC100 (AFS95065.1), CUC1 (AT3G15170), CUC2 (AT5G53950), CUC3 (AT1G76420), VND1 (AT2G18060), VND2 (AT4G36160), VND3 (AT5G66300), VND4 (AT1G12260), VND5 (AT1G62700). (*B*) Alignment of the deduced amino acid sequence of RhNAP with those of NAP proteins from other plant species.

**Additional file 2: Figure S2.** Transcriptional activation of RhNAP. Transcriptional regulation activity assays in protoplasts. GAL4-BD, vector control; GAL4-BD-RhNAPF, GAL4-BD-RhNAPN and RhNAPC represent full length, N-terminal and C-terminal of RhNAP fused to the GAL4-BD, respectively. Error bars indicate SE (*n* = 6); Student’s *t*-test, *P < 0.05, **P < 0.01.

**Additional file 3: Figure S3.** Analysis of the RhCKX6 protein sequence. Phylogenetic analysis of RhCKX6 together with known CKX family proteins. The phylogenetic tree file was produced by MEGA 5.2. Bootstrap values indicate the divergence of each branch and the scale indicates branch length. Accession numbers are as follows: RhCKX6 (JK618028), AtCKX1 (At2g41510), AtCKX2 (At2g19500), AtCKX3 (At5g56970), AtCKX4 (At4g29740), AtCKX5 (At1g75450), AtCKX6 (At3g63440), AtCKX7 (At5g21482), OsCKX1 (LOC_Os01g09260), OsCKX6 (LOC_Os02g12770), OsCKX7 (LOC_Os02g12780), ZmCKX1 (NP_001105591.1), ZmCKX6 (ADP38082.1), TaCKX1 (ABH07114.1), FvCKX1 (XP_004305707.1), FvCKX6 (XP_004303072.1), VvCKX1 (XP_002284560.1), VvCKX6 (XP_002270841.1), SlCKX2 (NP_001244909.1), SlCKX5 (NP_001244907.1), SlCKX7 (NP_001244908.1), MtCKX (XP_003599606.1) PhCKX (BAK52671.1), NtCKX7 (AII20187.1).

**Additional file 4: Figure S4.** qRT-PCR analysis of the expression of *RhNAP* and *RhCKX* genes in rose petals in response to exogenous ABA. Rose flowers at opening stage 2 were analyzed after 24 h of 100 μM ABA treatment. Control samples were treated with 0.05% EtOH without phytohormones for 24 h. *RhUBI1* was used as an internal control. All data shown are means ± standard deviation (*n* = 3); Student’s *t*-test, *P < 0.05, **P < 0.01.

**Additional file 5: Figure S5.** Drought tolerance and gene expression of *RhNAP* and *RhCKX6* overexpressing *A. thaliana* lines*.* (*A*) RT-PCR was conducted with fully expanded leaves of transgenic *A. thaliana* plants. *ACTIN2* was used as an internal control. (*B*) Tolerance of *RhNAP*- or *RhCKX6*-overexpressing *A. thaliana* plants to drought. T3 homozygous transformants were used in this experiment. 0 day drought, 14-day old well-watered plants; 15 days drought, 15 days after withholding water; 3 days rewatering, 3 days after rewatering; Survival rate was calculated from three independent experiments (~ 40 plants per line in one experiment).

**Additional file 6: Figure S6.** Effects of exogenous 6-benzylaminopurine (6-BA), *trans*-zeatin (*t*Z), isopentenyladenine (iP) and lovastatin on the senescence of rose petal discs. After 24 h pre-treatment with combinations of 100 μM 6-BA, 10 μM *t*Z, 10 μM iP, and 20 μM lovastatin, the petal discs were kept in water and photographed on days 7 and 13.

**Additional file 7: Figure S7.** Phenotypic analysis of *RhNAP* and *RhCKX6* overexpression lines. (*A*) Phenotypes of age-matched plants (approximately 35 days after germination, DAG) of wild type (WT), *RhNAP* and *RkCKX6*. (*B*) Phenotypes of leaves detached from the age-matched 40 DAG plants in *A*.

**Additional file 8: Figure S8.** Analysis of *RhCKX* gene expression. Expression of *RhCKX1* and *RhCKX7* in rose petals in response to dehydration treatment (*A*) and various opening stages (*B*). The internal control used was *RhUBI1*. (C) qRT-PCR analysis of *RhCKX* gene expression in rose petals in response to exogenous 6-BA. Rose flowers at opening stage 2 were analyzed after 24 h of 100 μM 6-BA treatment. Control samples were treated with 0.1 M NaOH without phytohormones for 24 h. *RhUBI1* was used as an internal control. All data shown are means ± standard deviation (*n* = 3); Student’s *t*-test, *P < 0.05, **P < 0.01.

**Additional file 9: Figure S9.** Anatomical structure of a petal viewed by scanning electron microscopy at flower full opening stage. (*A*, *B*) Adaxial epidermis; (*C*, *D*) Abaxial epidermis. Scale bar: 200 μm in *A* and *C*, magnification 200 x; 20 μm in *B* and *D*, magnification 2000 x.

**Additional file 10: Table S1.** Primers used in this study.

## Data Availability

Not applicable.

## Abbreviations

ABA: Abscisic acid
CKs: Cytokinins
CKX: Cytokinin oxidase/dehydrogenase
DAG: Days after germination
EMSA: Electrophoresis Mobility Shift Assay
GAD: GAL4 activation domain
GFP: Green fluorescent protein
GST: Glutathione S-transferase
NAP: *NAC-LIKE, ACTIVATED BY AP3/PI*
ORF: Open reading frame
PCD: Programmed cell death
PP2C: Protein phosphatase 2C
SAGs: Senescence associated genes
TFs: Transcription factors
TRV: Tobacco rattle virus
VIGS: Virus-induced gene silencing
