# Supplementary figures and images for: Regulation of rose petal dehydration tolerance and senescence by RhNAP transcription factor via the modulation of cytokinin catabolism
Source: Mol Hortic. 2021 Oct 11;1:13. doi: 10.1186/s43897-021-00016-7 (PMC10515265; doi:10.1186/s43897-021-00016-7)

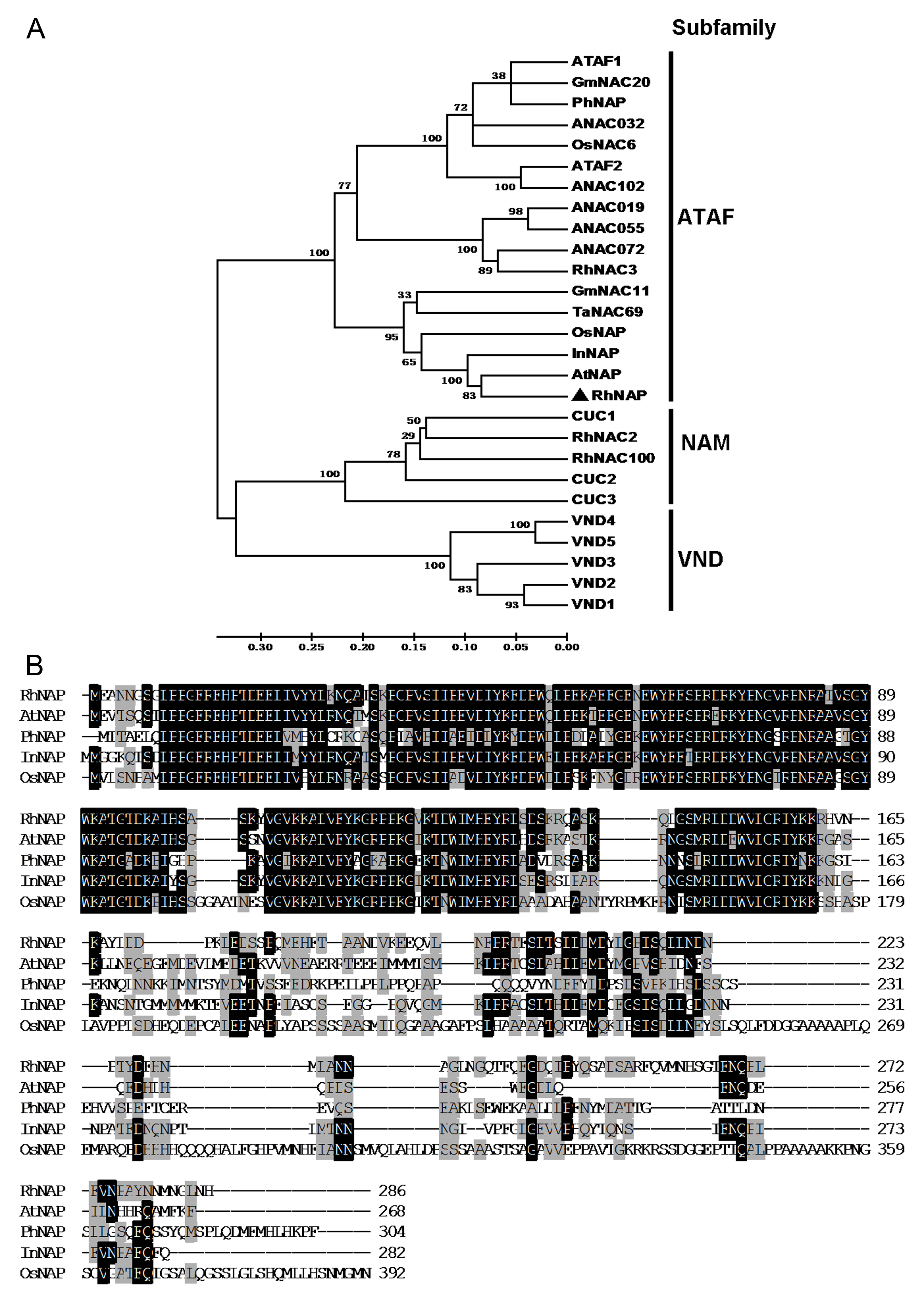

Supplement: Supplementary file 1 — Additional file 1: Figure S1. Analysis of the RhNAP protein sequence. (A) Phylogenetic analysis of RhNAP together with known NAC family proteins. The phylogenetic tree file was produced by MEGA 5.2. Bootstrap values indicate the divergence of each branch and the scale indicates branch length. Accession numbers are as follows: ATAF1 (AT1G01720), ATAF2 (AT5G08790), ANAC019 (AT1G52890), ANAC032 (AT1G77450), ANAC055 (AT3G15500), ANAC072 (AT4G27410), ANAC102 (AT5G63790), OsNAC6 (BAA89800), TaNAC69 (AAY44098.1), GmNAC11 (ACC66315.1), GmNAC20 (ACC66314), AtNAP (AT1G69490), PhNAP (AAM34773), InNAP (AB639146), OsNAP (LOC_Os03g21060), RhNAP (JK619941), RhNAC2 (JK619963), RhNAC3 (JK617768), RhNAC100 (AFS95065.1), CUC1 (AT3G15170), CUC2 (AT5G53950), CUC3 (AT1G76420), VND1 (AT2G18060), VND2 (AT4G36160), VND3 (AT5G66300), VND4 (AT1G12260), VND5 (AT1G62700). (B) Alignment of the deduced amino acid sequence of RhNAP with those of NAP proteins from other plant species. [file 43897_2021_16_MOESM1_ESM.tif]

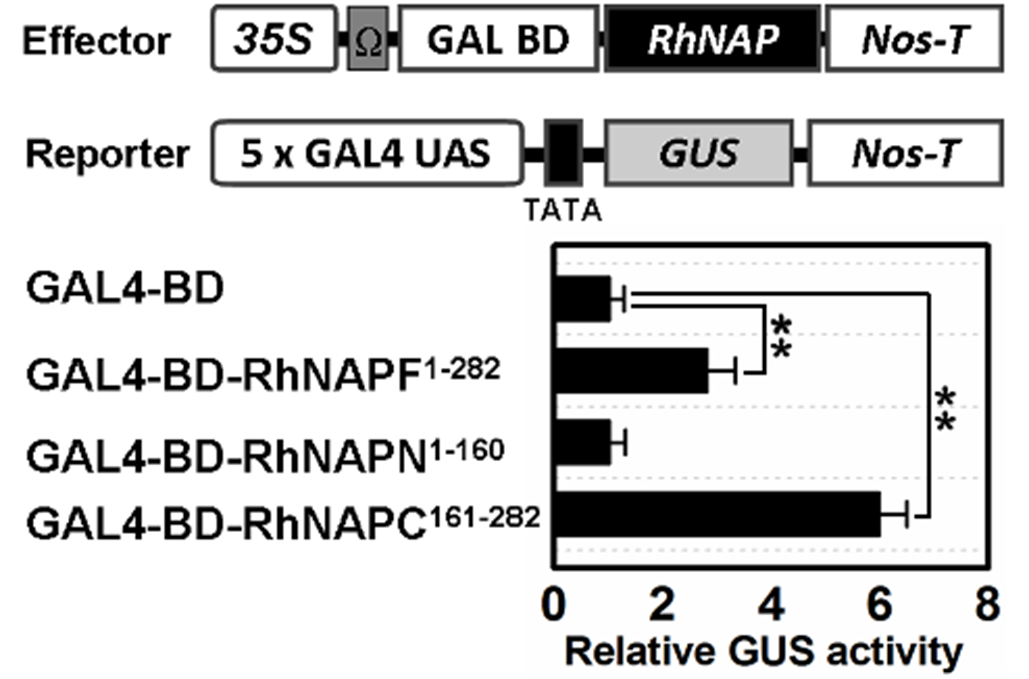

Supplement: Supplementary file 2 — Additional file 2: Figure S2. Transcriptional activation of RhNAP. Transcriptional regulation activity assays in protoplasts. GAL4-BD, vector control; GAL4-BD-RhNAPF, GAL4-BD-RhNAPN and RhNAPC represent full length, N-terminal and C-terminal of RhNAP fused to the GAL4-BD, respectively. Error bars indicate SE (n = 6); Student’s t-test, *P < 0.05, **P < 0.01. [file 43897_2021_16_MOESM2_ESM.tif]

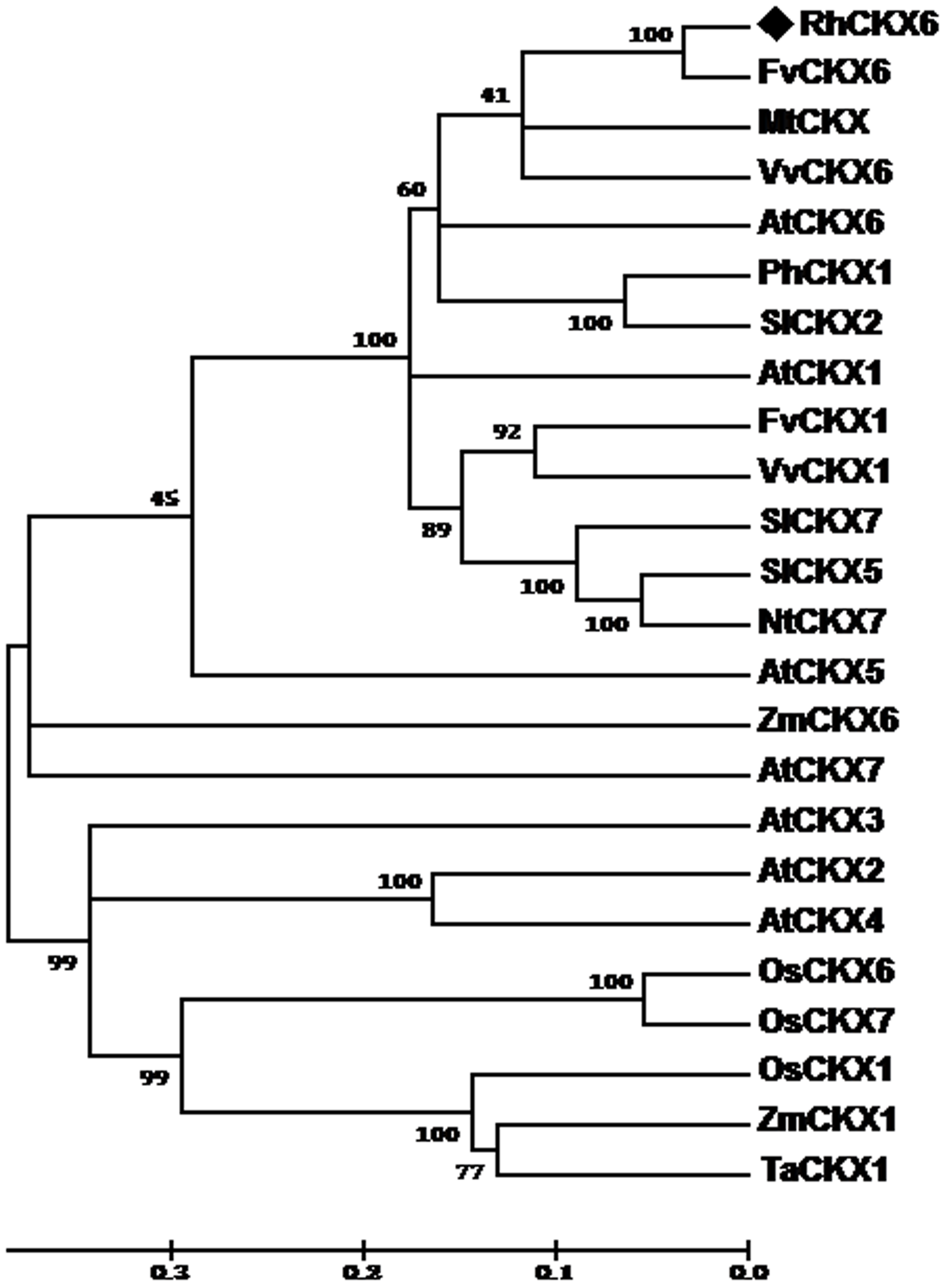

Supplement: Supplementary file 3 — Additional file 3: Figure S3. Analysis of the RhCKX6 protein sequence. Phylogenetic analysis of RhCKX6 together with known CKX family proteins. The phylogenetic tree file was produced by MEGA 5.2. Bootstrap values indicate the divergence of each branch and the scale indicates branch length. Accession numbers are as follows: RhCKX6 (JK618028), AtCKX1 (At2g41510), AtCKX2 (At2g19500), AtCKX3 (At5g56970), AtCKX4 (At4g29740), AtCKX5 (At1g75450), AtCKX6 (At3g63440), AtCKX7 (At5g21482), OsCKX1 (LOC_Os01g09260), OsCKX6 (LOC_Os02g12770), OsCKX7 (LOC_Os02g12780), ZmCKX1 (NP_001105591.1), ZmCKX6 (ADP38082.1), TaCKX1 (ABH07114.1), FvCKX1 (XP_004305707.1), FvCKX6 (XP_004303072.1), VvCKX1 (XP_002284560.1), VvCKX6 (XP_002270841.1), SlCKX2 (NP_001244909.1), SlCKX5 (NP_001244907.1), SlCKX7 (NP_001244908.1), MtCKX (XP_003599606.1) PhCKX (BAK52671.1), NtCKX7 (AII20187.1). [file 43897_2021_16_MOESM3_ESM.tif]

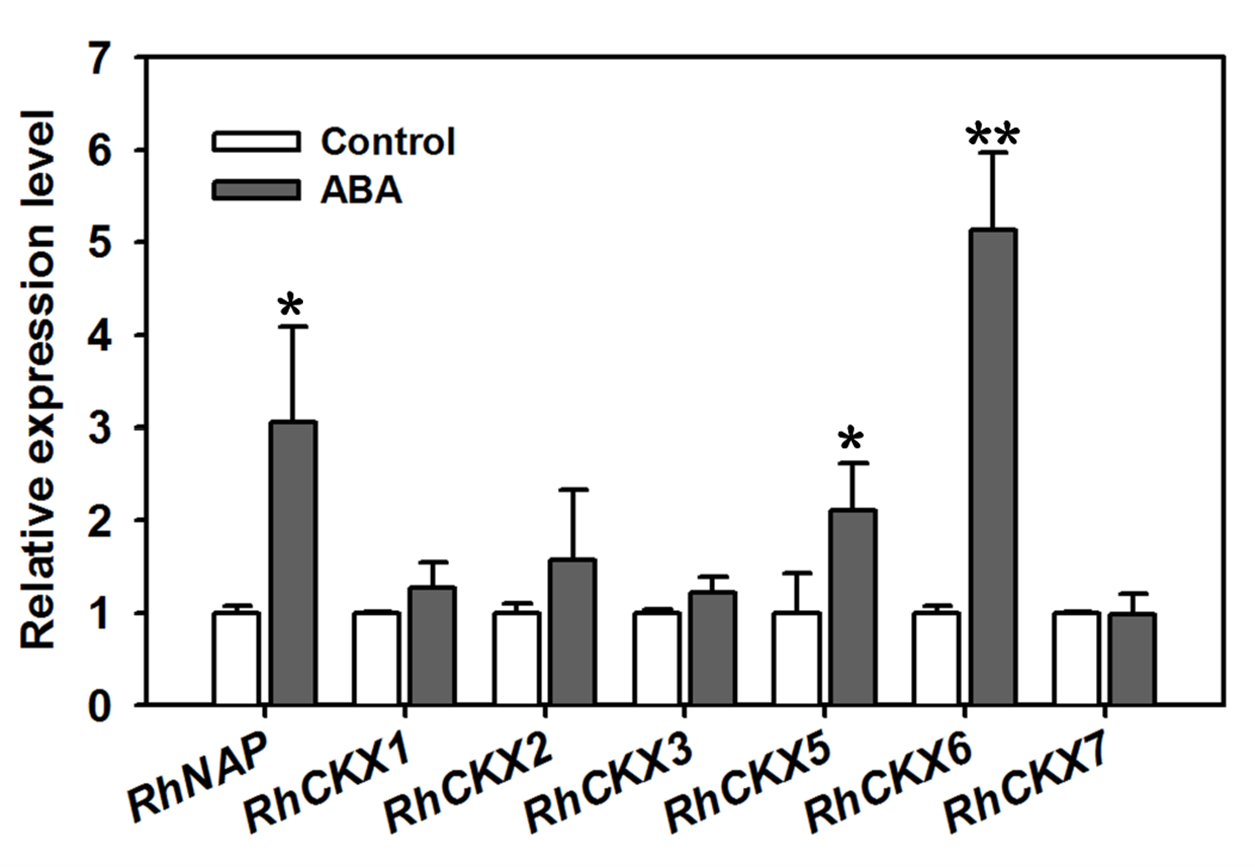

Supplement: Supplementary file 4 — Additional file 4: Figure S4. qRT-PCR analysis of the expression of RhNAP and RhCKX genes in rose petals in response to exogenous ABA. Rose flowers at opening stage 2 were analyzed after 24 h of 100 μM ABA treatment. Control samples were treated with 0.05% EtOH without phytohormones for 24 h. RhUBI1 was used as an internal control. All data shown are means ± standard deviation (n = 3); Student’s t-test, *P < 0.05, **P < 0.01. [file 43897_2021_16_MOESM4_ESM.tif]

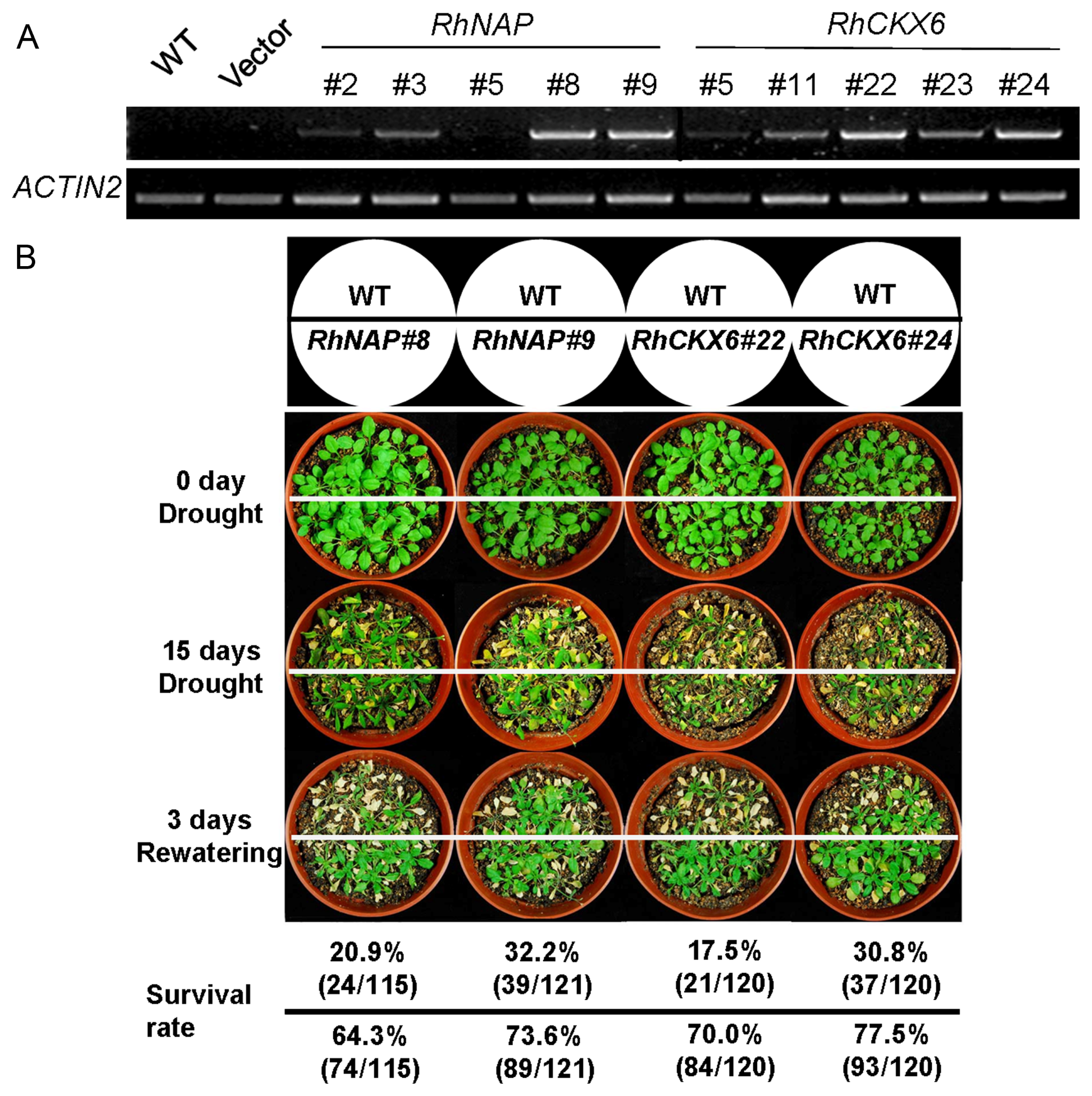

Supplement: Supplementary file 5 — Additional file 5: Figure S5. Drought tolerance and gene expression of RhNAP and RhCKX6 overexpressing A. thaliana lines. (A) RT-PCR was conducted with fully expanded leaves of transgenic A. thaliana plants. ACTIN2 was used as an internal control. (B) Tolerance of RhNAP- or RhCKX6-overexpressing A. thaliana plants to drought. T3 homozygous transformants were used in this experiment. 0 day drought, 14-day old well-watered plants; 15 days drought, 15 days after withholding water; 3 days rewatering, 3 days after rewatering; Survival rate was calculated from three independent experiments (~ 40 plants per line in one experiment). [file 43897_2021_16_MOESM5_ESM.tif]

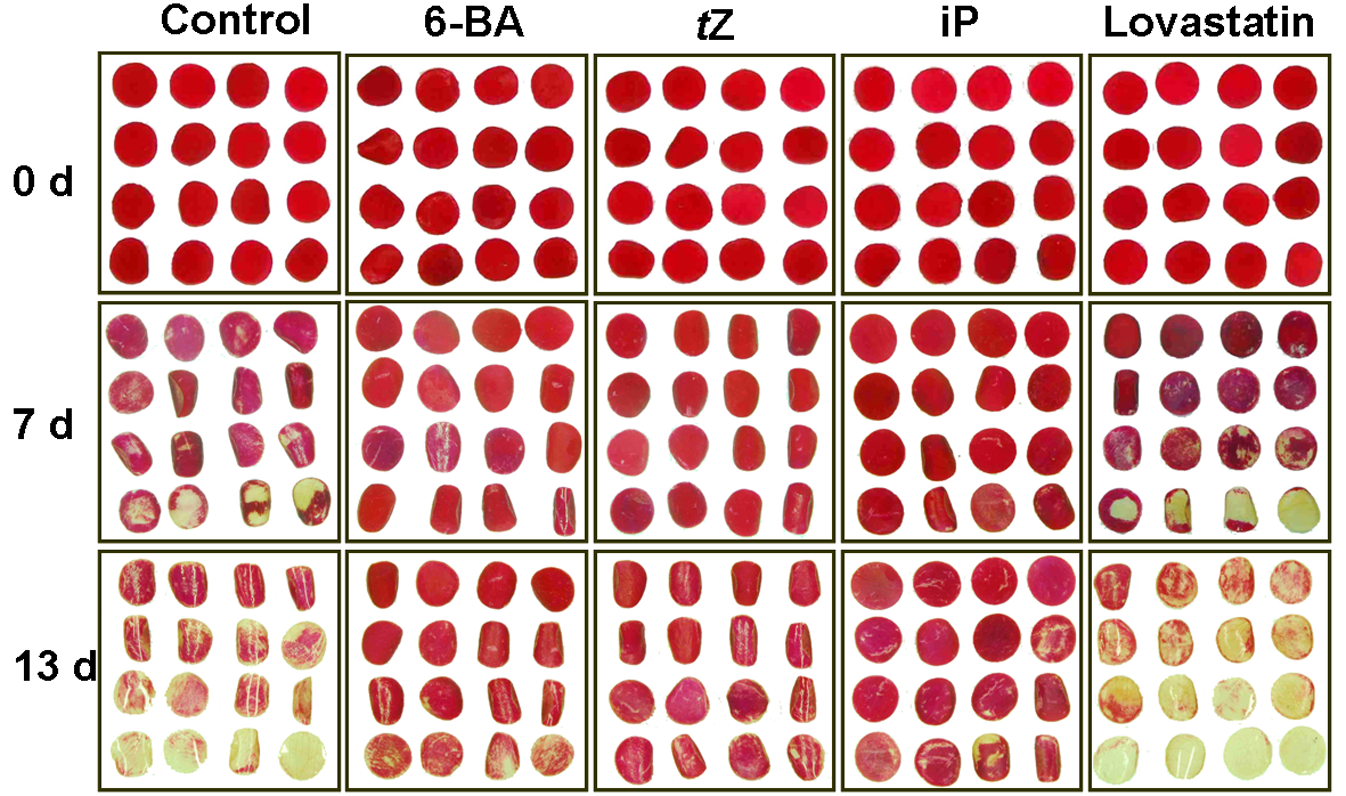

Supplement: Supplementary file 6 — Additional file 6: Figure S6. Effects of exogenous 6-benzylaminopurine (6-BA), trans-zeatin (tZ), isopentenyladenine (iP) and lovastatin on the senescence of rose petal discs. After 24 h pre-treatment with combinations of 100 μM 6-BA, 10 μM tZ, 10 μM iP, and 20 μM lovastatin, the petal discs were kept in water and photographed on days 7 and 13. [file 43897_2021_16_MOESM6_ESM.tif]

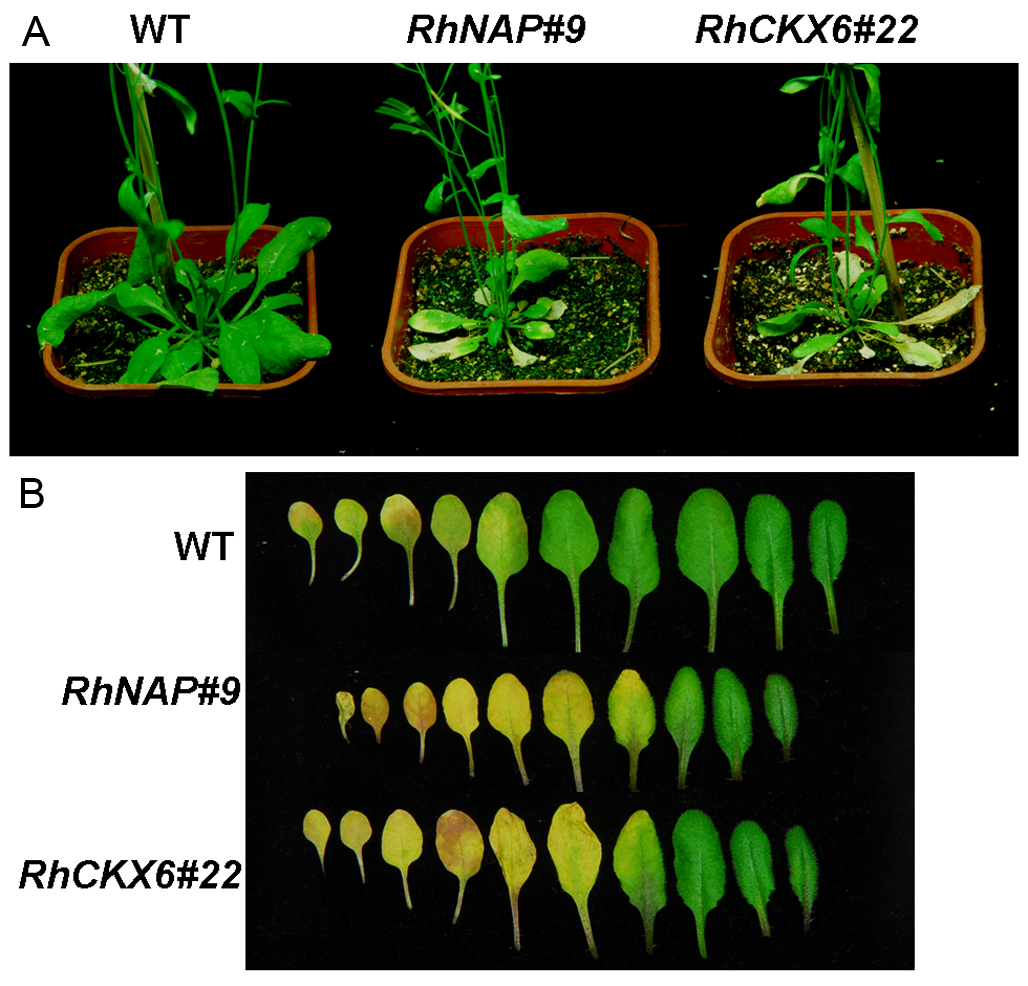

Supplement: Supplementary file 7 — Additional file 7: Figure S7. Phenotypic analysis of RhNAP and RhCKX6 overexpression lines. (A) Phenotypes of age-matched plants (approximately 35 days after germination, DAG) of wild type (WT), RhNAP and RkCKX6. (B) Phenotypes of leaves detached from the age-matched 40 DAG plants in A. [file 43897_2021_16_MOESM7_ESM.tif]

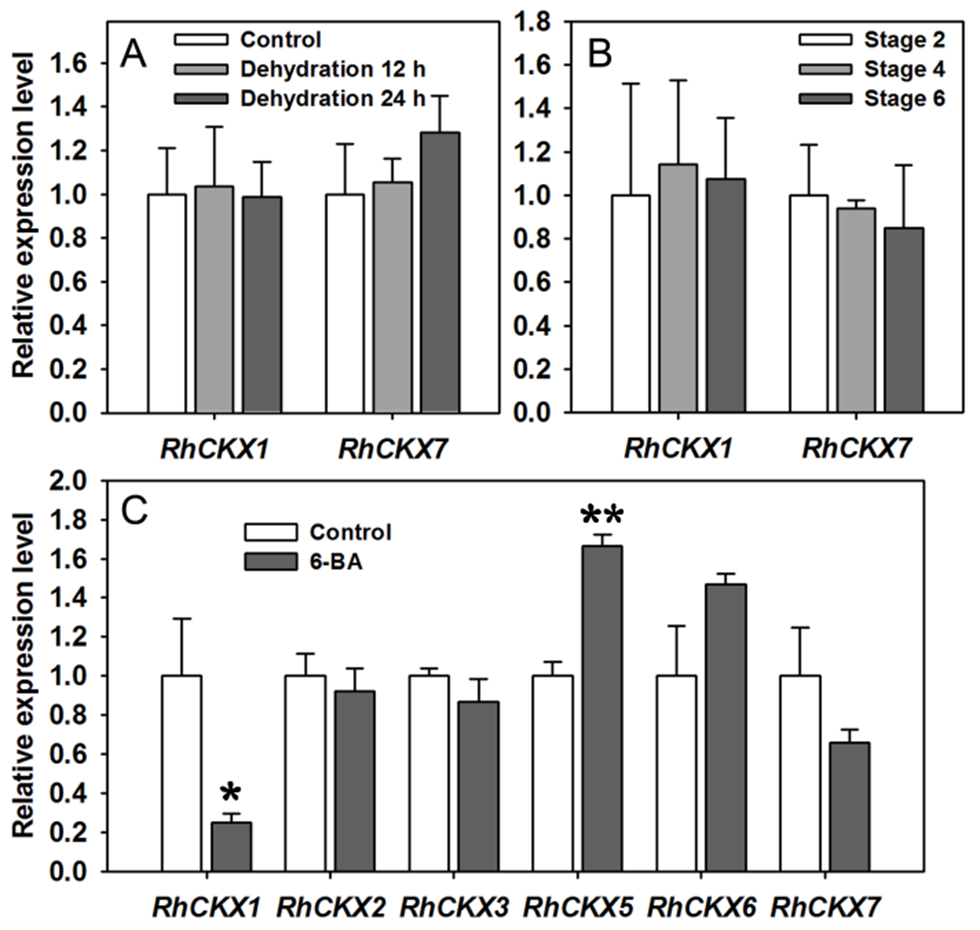

Supplement: Supplementary file 8 — Additional file 8: Figure S8. Analysis of RhCKX gene expression. Expression of RhCKX1 and RhCKX7 in rose petals in response to dehydration treatment (A) and various opening stages (B). The internal control used was RhUBI1. (C) qRT-PCR analysis of RhCKX gene expression in rose petals in response to exogenous 6-BA. Rose flowers at opening stage 2 were analyzed after 24 h of 100 μM 6-BA treatment. Control samples were treated with 0.1 M NaOH without phytohormones for 24 h. RhUBI1 was used as an internal control. All data shown are means ± standard deviation (n = 3); Student’s t-test, *P < 0.05, **P < 0.01. [file 43897_2021_16_MOESM8_ESM.tif]

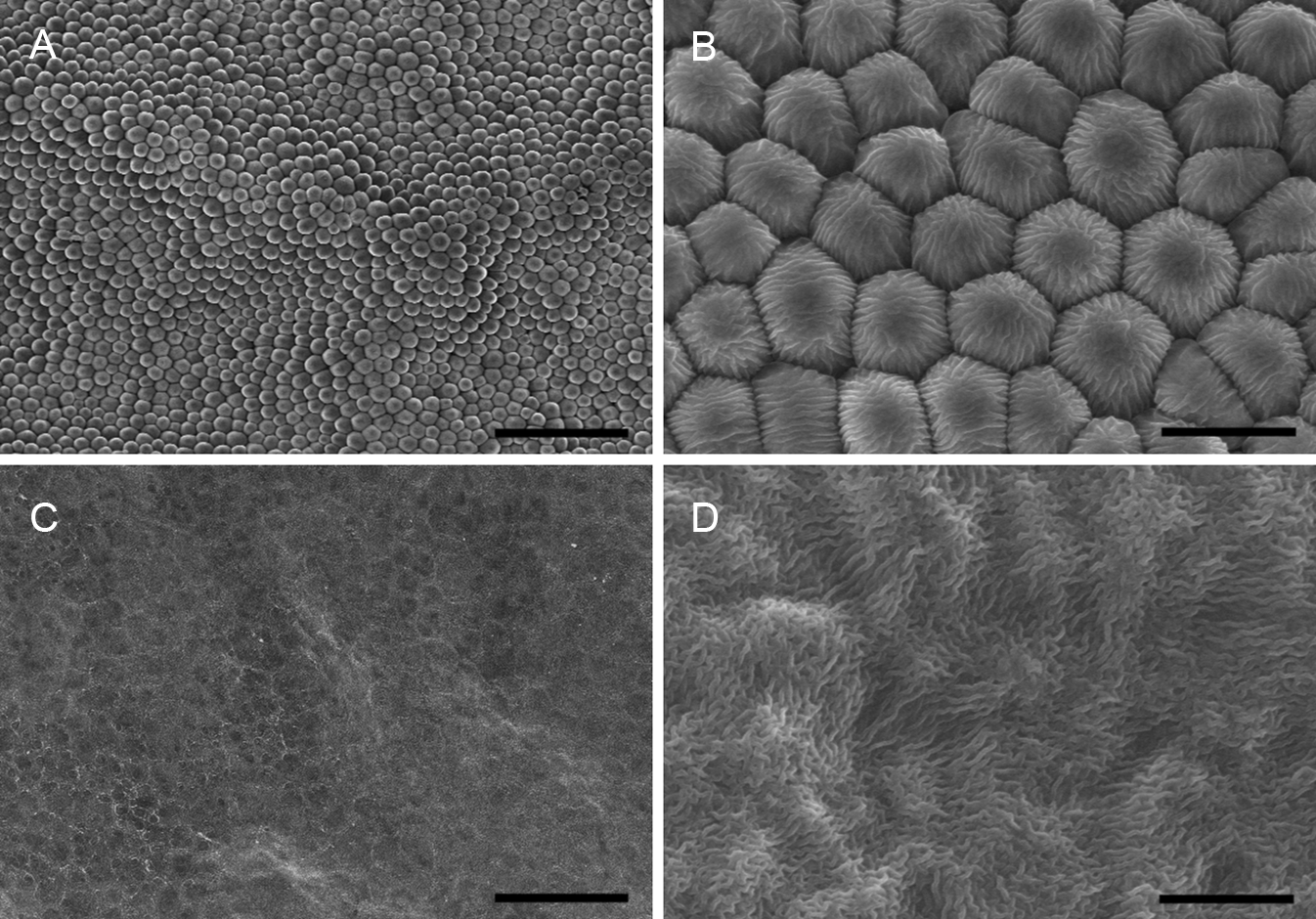

Supplement: Supplementary file 9 — Additional file 9: Figure S9. Anatomical structure of a petal viewed by scanning electron microscopy at flower full opening stage. (A, B) Adaxial epidermis; (C, D) Abaxial epidermis. Scale bar: 200 μm in A and C, magnification 200 x; 20 μm in B and D, magnification 2000 x. [file 43897_2021_16_MOESM9_ESM.tif]
