# Supplementary material for: Regulation of rose petal dehydration tolerance and senescence by RhNAP transcription factor via the modulation of cytokinin catabolism
Source: Mol Hortic. 2021 Oct 11;1:13. doi: 10.1186/s43897-021-00016-7 (PMC10515265; doi:10.1186/s43897-021-00016-7)
Supplement: Supplementary file 10 — Additional file 10: Table S1. Primers used in this study. [file 43897_2021_16_MOESM10_ESM.docx]

**Table S1.** Primers used in this study.

|  | Forward primers | Reverse primers |
| --- | --- | --- |
| Primers used for qRT-PCR | | |
| RhNAP | TGGCTCTGGACTTCCTCCTGG | GGGCACGGCTTTGAAATGGCT |
| RhCKX1 | GCCACCCCAACCATTATTCC | ACTGCTGACGGTTGGAAATG |
| RhCKX2 | AGACGGTCGGATTATTGCTCA | TTGGCGGAGCATTTCCTCTT |
| RhCKX3 | GGCCTGTCCTTTGCGTATCT | GCTGCATGAGAACGAGGGTA |
| RhCKX5 | GACGAGGGAGTGTTCAAGGG | GATCGCAGCAATGCCACAAG |
| RhCKX6 | GCCACCCCAACCATTATTCC | ACTGCTGACGGTTGGAAATG |
| RhCKX7 | CTATTGCGTGTGAAGCAGGC | CAGGTTTAGCCAGGGGTGAG |
| RU00527 | GGCATGCGCAAAATAGTGGT | ACTCCCCCTTGCTGTTTGAG |
| RU12149 | AAGCTACGAGAACCGTCCG | ATGATAGGGCTGACGTTGGG |
| RU39694 | AAGCTCGGAGGTTAAGCAGG | TCTTCCATGGTCTTGCGCTT |
| RU47281 | CTTGGAAGAAGGGGCTGAGG | ACTGTCAATGCTGGAAGAAGGT |
| RU60370 | TAAGCAGTGGCATGGTTGGT | AACCAGCCGGCGAGTTTAAT |
| RU23970 | TCGGTGAATCGGGCAAAGAA | GGCTCATGTCAGAATCATCCACT |
| RU03558 | CTAATTTGTCGGTCGACTCCC | ATGATCGGAGGAGAGAGGCA |
| RU01455 | ATCTGGGTAACACCGCAACC | TTGAAGGTTTCTCGGGGCAG |
| RU03861 | GCTTCTACCTCGGACCCTTT | TCTCTCTTTTGGGTCTGAAGC |
| RU04740 | TCCTACGCTTTCGATCATGTGT | ATCTGTTTCGGAGCCATTCA |
| RU22946 | AGCACTTTGGACCGTGGTAG | GTCCCTTCAGCCCTTTGACT |
| RU25535 | CAGGCACAGAGTCCCTCATC | GCGACTAGGAGGACTGATGC |
| RU07831 | AGTGTAGCATCCGCCTGTCT | TCCACAAGTCCGAGGAACCA |
| RU06450 | TGCGGAGTGAGTATCCTGAGA | TGATCTGGTTTCCATGTCCCA |
| RhSAG12 | AGCGGAGAAGCCTTTCAGTC | CAGCATGGTTCAGGCTGGTA |
| RhUBI1 | GGGCAATCATCTGGAATTGCTCGT | GCCCCCAAAGAGAAACCCTGCG |
| Primers used to generate DNA constructs | | |
| TRV-RhNAP | CTGGATCCGGACCAATTTCCCAGCTTTTG | CGACTCGAGCGGATTGAAATGAATTCATTTTC |
| TRV-RhCKX6 | CTGGATCCGAGGCCCTTAAGGGATGT | CGACTCGAGGAAACTGGATTCAAGAAATAT |
| RhNAPF-BD | ACCGTCGACATGGAGGCCAATAATGGCTCT | GCACTGCAGTTAGTGGTTCAAACCATTCATGTTG |
| RhNAPN-BD | ACCGTCGACATGGAGGCCAATAATGGCTCT | GCACTGCAGCTTCTTATAGATCCTACACAGGACCC |
| RhNAPC-BD | CGGAATTCAGGCATGTCAACAAGGCTTACT | GCACTGCAGTTAGTGGTTCAAACCATTCATGTTG |
| Super-RhNAP-GFP | ACCAAGCTTATGGAGGCCAATAATGGCTCT | CAGCTGCAGGTGGTTCAAACCATTCATGTTGTT |
| pGEX-RhNAP | GCGGATCCATGGAGGCCAATAATGGCTCT | GCGAATTCTTAGTGGTTCAAACCATTCATGTTG |
| RhCKX6p-GUS | GCACTGCAGTACTGTGGGAGATAAGTGACAAG | GTGGATCCTTTCGGTTAGAAGATATGGAGG |
| GAD-RhNAP | CGGAATTCATGGAGGCCAATAATGGCTCT | GCACTCGAGTTAGTGGTTCAAACCATTCATGTTG |
| RhCKX6p-LacZ | AATTCTCATCTGATCATTCACGTGCGCAAGACATG | TCGACATGTCTTGCGCACGTGAATGATCAGATGAG |
| RhCKX6pm-LacZ | AATTCTCATCTGATCCGGACATGTCGCAAGACATG | TCGACATGTCTTGCGACATGTCCGGATCAGATGAG |
